# Low Temperature Stress Tolerance: An Insight Into the Omics Approaches for Legume Crops

**DOI:** 10.3389/fpls.2022.888710

**Published:** 2022-06-03

**Authors:** Kaisar Ahmad Bhat, Reetika Mahajan, Mohammad Maqbool Pakhtoon, Uneeb Urwat, Zaffar Bashir, Ali Asghar Shah, Ankit Agrawal, Basharat Bhat, Parvaze A. Sofi, Antonio Masi, Sajad Majeed Zargar

**Affiliations:** ^1^Proteomics Laboratory, Division of Plant Biotechnology, Sher-e-Kashmir University of Agricultural Sciences and Technology of Kashmir (SKUAST-K), Shalimar, India; ^2^Department of Biotechnology, School of Biosciences and Biotechnology, Baba Ghulam Shah Badshah University, Rajouri, India; ^3^Department of Life Sciences, Rabindranath Tagore University, Bhopal, India; ^4^Deparment of Microbiology, University of Kashmir, Srinagar, India; ^5^Division of Animal Biotechnology, Sher-e-Kashmir University of Agricultural Sciences and Technology of Kashmir, Srinagar, India; ^6^Division of Genetics and Plant Breeding, Sher-e-Kashmir University of Agricultural Sciences and Technology of Kashmir, Srinagar, India; ^7^Department of Agronomy, Food, Natural Resources, Animals, and Environment, University of Padova, Padua, Italy

**Keywords:** cold stress, omics approaches, legumes, tolerance, transcriptional factors, proteomics

## Abstract

The change in climatic conditions is the major cause for decline in crop production worldwide. Decreasing crop productivity will further lead to increase in global hunger rate. Climate change results in environmental stress which has negative impact on plant-like deficiencies in growth, crop yield, permanent damage, or death if the plant remains in the stress conditions for prolonged period. Cold stress is one of the main abiotic stresses which have already affected the global crop production. Cold stress adversely affects the plants leading to necrosis, chlorosis, and growth retardation. Various physiological, biochemical, and molecular responses under cold stress have revealed that the cold resistance is more complex than perceived which involves multiple pathways. Like other crops, legumes are also affected by cold stress and therefore, an effective technique to mitigate cold-mediated damage is critical for long-term legume production. Earlier, crop improvement for any stress was challenging for scientific community as conventional breeding approaches like inter-specific or inter-generic hybridization had limited success in crop improvement. The availability of genome sequence, transcriptome, and proteome data provides in-depth sight into different complex mechanisms under cold stress. Identification of QTLs, genes, and proteins responsible for cold stress tolerance will help in improving or developing stress-tolerant legume crop. Cold stress can alter gene expression which further leads to increases in stress protecting metabolites to cope up the plant against the temperature fluctuations. Moreover, genetic engineering can help in development of new cold stress-tolerant varieties of legume crop. This paper provides a general insight into the “omics” approaches for cold stress in legume crops.

## Introduction

In addition to the expanding population rate, increased biotic and abiotic pressures and limited agricultural land availability are significant restraints for farming and food production. Today, the most important concern for mankind is ensuring food security for an expanding population. It is well-known fact that the world population is increasing exponentially and believed to reach ten billion by 2050, needing a 60–100% increase in global food production (FAOSTAT, 2021). In one or other way, both these issues have adversely impacted agriculture sector. Crop yield and production of a particular crop is affected by numerous abiotic and biotic factors. Legumes are one of the essential staple foods after cereal crop. More than 1,300 legume species are grown worldwide out of which only 20 legume species are consumed by humans, because of its high protein, fiber, carbohydrate, and low-fat content (Câmara et al., 2013). Legumes like common bean, pea, and soybean are great source of proteins, micronutrients, dietary fibers, and carbohydrates for humans as well as animals (Zargar et al., 2017). Furthermore, legumes can serve as a great source of animal feed and can help in improvement of soil as it fixes atmospheric nitrogen in the soil (Meena and Lal, 2018). Since late last century, legume production has declined due to various environmental, socio-economic, and genetic factors. Various types of abiotic stresses *viz* drought, extreme temperature, salinity, and heavy metal stress have declined the legume production by approximately 50% worldwide (Jan et al., 2022). Cold stress alone has resulted in a decline of the overall productivity of legume crops, such as Chickpea and soybean by ~60% and mungbean by ~70% in recent years (Jan et al., 2022). Extreme temperature (either cold or heat) is one of the most important factors affecting overall developmental stages of plant and crop phenology that leads to loss in productivity and yield (Repo et al., 2008). Cold stress is differentiated on the basis of degree of temperature and is categorized into chilling (0–15°C) or freezing (<0°C) stress. Cold stress primarily affects the plasma membrane of the cell resulting in leakage of different types of ions, proteins, and lipids (Cheng et al., 2010). Physiological as well as cellular perturbations occur in a variety of legume species after encountering cold stress. Plants must retain cell behavior and activity under cold stress, especially the stability of the cell membrane and the structure of the protein with biological activity, in order to survive harsh settings. Earlier developing stress-tolerant varieties *via* conventional breeding was laborious and time-consuming process which was eased down by the new modern breeding approaches as well as by multi-omic techniques that simplify the improvement and development of new cold stress-tolerant varieties in various crops. Thus, to survive the cold stress, plants adapt different approaches which include gene expression, reprogramming, and alteration in different metabolic processes which help in modulation of various proteins of stress induction (Jan et al., 2019). In-depth knowledge of how plants react to cold stress could provide critical information and biological resources for improving crop cold-stress tolerance. Because of multigenicity, understanding abiotic stress tolerance, without breeding consideration had proven difficult which resulted in non-existence of traditional crop line that has cumulative tolerance for complex trait of drought and high salinity stress. Many plant biologists are of the opinion that to decipher the tolerance pathway mechanisms by understanding correlative evidence from different plant species rely on biophysical and biochemical mechanisms that regulate stress tolerance (Levitt, 1980). These discoveries offered guiding concepts for progressing from phenotype to protein and enzyme analysis, gene structure, and gene expression investigations, leading to the production as well as analysis of transgenic and mutant species. Here, in this review, we have summarized different available multi-omic techniques used for identifying the cold tolerance genes/protein and to study the role of different cold-responsive proteins in legume plants.

## Impact of Cold Stress in Legumes

**Figure 1:**
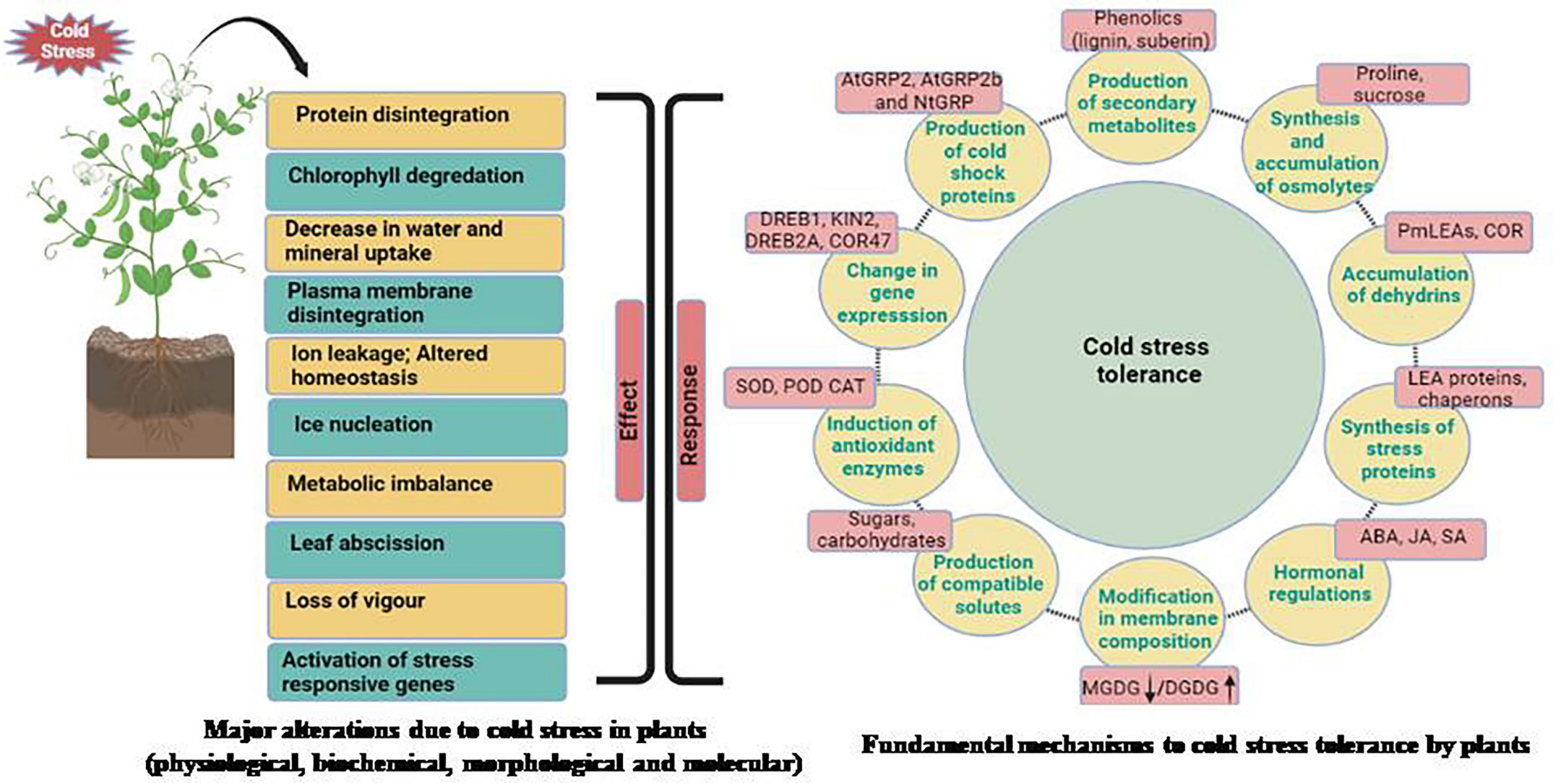
Impact of cold stress at morphological, physiological, biochemical, and molecular levels and different mechanisms adapted by plants for combating cold stress.

**Figure 2:**
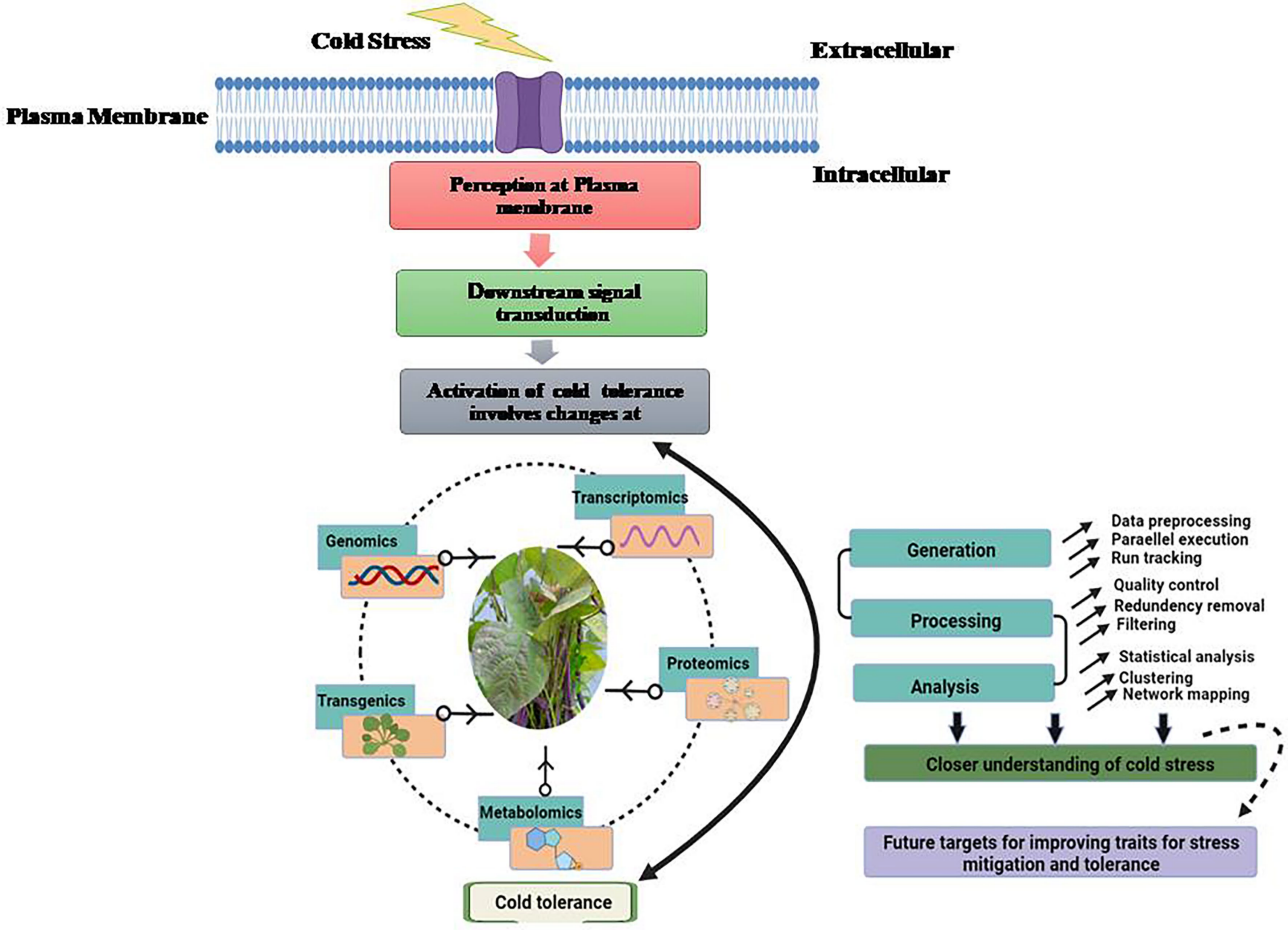
Multi-omic approaches for cold stress tolerance involving analysis of cold stress perception to downstream signaling and data processing with analysis and future targets for improving traits for cold stress.

For appropriate growth and development, each plant requires a specific set of temperatures. For example, a set of temperature ideal for one plant may be stressful for another. Plants native to warm settings have been seen to show damage signs when exposed to low temperatures. Plants like soybean (*Glycine max*), show indications of harm when exposed to temperatures below 10–15°C (Maqbool et al., 2010). The emergence of damage signs, on the other hand, is dependent on a plant’s sensitivity to cold stress and differs from plant to plant. Plants are affected by stressful conditions at all stages of development. Unfavorable temperatures can have a direct impact on seed germination and emergence, as well as early survival and growth of seedlings (Maqbool et al., 2010). For example, cold stress during germination in chickpea increased susceptibility to soil-borne diseases, as well as poor crop establishment and even seedling death. Cold stress causes phenotypic changes in plants, such as reduced leaf expansion, wilting, and chlorosis (leaf yellowing), which can lead to necrosis (death of tissue). Plants reproductive development is also harmed by cold stress (Shafiq et al., 2012). Poor germination, stunted seedlings, yellowing of leaves, wilting, and diminished tillering are all symptoms of cold stress. The effects of cold stress on plants at the reproductive stage delay heading and result in pollen sterility, which is regarded to be one of the main causes of crop production loss (Suzuki et al., 2008). The most serious side effect of cold stress in plant has been plasma membrane damage. This has been documented as a result of dehydration caused by cold stress. Plasma membranes become more static at low temperatures, limiting fluidity. As a result, the membrane becomes more rigid and may lose its function (Sanghera et al., 2011; Hatfield and Prueger, 2015). Rupturing of plasma membrane and tonoplast resulted in solute leakage. Cold stress, when combined, causes a loss of membrane integrity, which leads to solute leakage (Maqbool et al., 2010). The research of phase transitions in mungbean cell membranes was hailed as a breakthrough in the field of legumes. Increased electrolyte leakage in five-day-old seedlings exposed to cold temperature (4°C) caused irreversible chilling harm. Furthermore, cold stress compromises the integrity of intracellular organelles, resulting in compartmentalization loss. Plants that are subjected to cold stress have a reduction in photosynthesis, protein synthesis, and general metabolic functions. Different physiological, morphological, biochemical and molecular changes take place when a particular plant faces stress condition (Figure 1). Multi-omic approaches for understanding cold stress tolerance involves analysis of stress perception to downstream signaling and data processing for improving traits to develop legumes with better cold tolerance (Figure 2).

## Genomics: A Key to Understand QTLs/MTA Associated With Cold Stress Tolerance

Genomics deals with study of genetic variation as well as identification of novel QTLs/trait-specific genes *via* different genetic techniques to generate superior cultivars. The availability of genome sequence of a particular crop helps in identification of quantitative trait genes which ultimately leads to crop improvement. Till date, genome sequence of 15 legume crops are available in database. Table 1 summarizes different legume species with respective genome sizes. The discovery of candidate genes associated with many quantitative traits particular to crops pave a way for identification and selection of superior lines that can further be used in marker-assisted selection (MAS), Genome Assisted Breeding (GAB), and other breeding programs in agricultural crops (Jaganathan et al., 2020). Development of mapping population (RILs, NILs, MAGIC) and diverse germplasm of cold stress tolerance in legume crops could be important for improving the legume breeding. PCR and hybridization-based molecular markers are utilized for identification of QTLs and candidate gene in various biotic and abiotic stress in crops. In case of cold tolerance in legumes, not much work has been done so far. Thus to understand the impact of cold stress on different parameters at genetic level there is need to identify QTLs/genes associated with these parameters.

**Table 1:** Available genome size of various legumes along with scientific name.

**S. No.**	**Common name**	**Scientific name**	**Genome size (GB)**	**Reference**
1	Senna	Alexandrian senna	1.76	Macas et al., 2015
2	Gum arabic	Senegalia senegal	1.47	Macas et al., 2015
3	Lupinus	Lupinus polyphyllus	0.90	Macas et al., 2015
4	Ground nut	Arachis hypogaea	1.74	Chang et al., 2018
5	Common bean	Phaseolus vulgaris	0.59	Macas et al., 2015
6	Mungbean	Vigna radiata	0.52	Macas et al., 2015
7	Soybean	Glycine max	1.10	Schmutz et al., 2010
8	Pigeon pea	Cajanus cajan	0.86	Singh et al., 2012
9	Sweet clover	Melilotus officinalis	1.10	Macas et al., 2015
10	Clover	Trifolium	0.96	Macas et al., 2015
11	Barrel medic	Medicago truncatula	0.47	Macas et al., 2015
12	Garden pea	Pisum sativum	4.36	Macas et al., 2015
13	Broad bean	Vicia faba	13.06	Macas et al., 2015
14	Black locust	Robinia pseudoacacia	0.64	Macas et al., 2015
15	Birdsfoottrefoil	Lotus japonicus	0.47	Sato et al., 2008

## QTLs Contributing to Cold Stress Tolerance

Advances in phenomics and genome sequencing molecular breeding techniques are potential tools for crop development but cost-effectiveness of such approaches restricts their use. SSR markers were frequently employed in rice to identify QTLs in stress and gene flow programs (Kumar et al., 2014). Applying SNP markers in a layered aided linkage map, many climate QTLs in maize were discovered (Li et al., 2016). For legume crops, various markers have been used to study genetic diversity and other genetic level research for crop improvement program or for development of smart climate resilience crops. Various QTLs/SNPs identified in different legumes under cold stress are tabulated in Table 2. In chickpea, very less genomic resources are studied on cold stress (Jha et al., 2020). SSR markers have been combined with DArT and CAPS markers to identify a favorable QTL for abiotic stress and effectively introgressed, leading to the release of superior chickpea varieties (Varshney et al., 2014; Jaganathan et al., 2020). Other legumes, such as peanuts and pigeonpea, have also benefited from genomic-assisted breeding for abiotic and biotic stress tolerance (Varshney et al., 2019). While completing QTL research in the population derived from ICC 4958 × PI 489777, QTLs for chilling resistance in six different environmental conditions were identified (Mugabe et al., 2019). Three QTLs were identified on LG1B, LG3, and LG8, as well as CT Ca-3.1 (on LG3) and CT Ca-8.1 (on LG8) of which one QTL was detected in single environment while other two QTLs were important for all six environments by using CIM QTL mapping (Mugabe et al., 2019). Regarding plant height and seed content, two QTLs were discovered (Ht Ca-4 was discovered on LG 4 with a LOD value of 6.5, and Ht Ca-8 on LG 8 with a LOD value of 6.5). Ht Ca-4 accounted for 20.21% of the variance, while Ht Ca-8 accounted for 19.97% of the polymorphism. However, two QTLs were discovered for seed ellipsoid volume, the first QTL, SEV Ca-1(A), with a LOD value of 7.4, was discovered on LG 1(A), representing 15.93% of phenotypic variance and SEV Ca-4, the second QTL, was discovered on LG 4 and has a LOD score of 11.8, explaining 29.41% of variability (Mugabe et al., 2019). Enhancing cold tolerance for production of winter period pea variety is a challenging task. When breeding cold-season varieties, chilling tolerance, as well as seed output and purity, along with genetic factors of cold/frost tolerance, genetic connections with developmental and yield characteristics must be taken into account (Klein et al., 2014). Groups of hybrid inbred lines were examined in six distinct climatic situations using a newly discovered basis of cold stress. A genomic map with 679 microsatellite tools spread over seven clusters and a total length of 947.1 cM was created. For all assessed variables, 186 QTLs were discovered, accounting for 9 to 71% of variations. Specific frost tolerance may be developed to increase seed growth and yield in winter pea crop (Klein et al., 2014). Cold stress has affected sugar concentration, leakage of electrolytes, osmotic pressure, and activity of ribulose 1,5 bisphosphate in pea plant. RILs developed from the cross of Champagne (frost tolerant) and Terese (frost sensitive) were used for the identification of chromosomal regions linked to frost tolerance (QTLs) associated with sugar content under frost condition (Dumont et al., 2009). Effect of Raffinose metabolism and RuBisCO activity have an essential role in acclimatizing cold stress in pea (Dumont et al., 2009).

**Table 2:** Various QTLs/SNPs identified in different legumes under cold stress that can be used in MAS/MAB for cold tolerance in legumes.

S.No.	Legume	Bi parental cross/diverse germplasm	Approach	QTL/SNP	Linked trait	Reference
1	Chickpea (*Cicer arietinum*)	ICC 4958 × PI 489777	QTL mapping	7 QTLs	Plant height and seed content	Mugabe et al., 2019
2	Pea (*Pisum sativum*)	China (JI1491) × Caméor	QTL mapping	161 QTLs	Internode length, branching type, hilum color, seed weight, harvest index and seed protein content.	Klein et al., 2014
		Champagne × Terese	QTL mapping	25 QTLs	Leakage of electrolytes,sugar concentration, osmotic pressure, and RuBisCO activity	Dumont et al., 2009
3	White clover (*Trifolium repens*)	192 diverse germplasm	GWAS	17 SNPs	Stolon dry weight, diameter, length, water soluble carbohydrate degradation rate, Petiole length, Leaf area, dry matter Annual dry matter	Inostroza et al., 2018
4	Pea (*Pisum sativum*)	365 diverse pea accessions	GWAS	62 SNPs	Frost damage (FD) loci	Beji et al., 2020
5	Sorghum [*Sorghum bicolor* (L.) Moench]	Chinese landrace ‘Shan Qui Red,’ (SQR, cold-tolerant) and SRN39 (cold-sensitive)	QTL mapping	2 QTLs	Germination	Knoll, 2008
		242 accesions from ICRISAT	GWAS	1 SNP	Low-temperature germination	Upadhyaya et al., 2016
6	*Medicago sativa* L.	3,010 x CW 1010 (F_1_ mapping population)	QTL mapping	20 QTLs	Visual rating-based FT, percentage survival (PS), control regrowth ratio (RR), and control biomass ratio (BR)	Adhikari et al., 2021
7	Winter faba bean	Côte d’Or 1 (French landrace), and BeanPureLine 4,628 (BPL4628, Chinese inbred line)	QTL mapping	17 QTLs	11 frost tolerant and physiological traits.	Sallam et al., 2016
		Gottingen Winter Bean population (GWBP)	GWAS	25 SNPs	Three traits AUSPC (after hardening), LTAF, and LCAF	Sallam et al., 2016

## Marker Trait Association (MTA): For Cold Tolerance

Marker–trait association (MTA) or Genome-wide association (GWAS) studies deal with association of desired trait and molecular markers across the genome of a particular crop. This technique has advantage over biparental mapping as it involves study of highly diverse germplasm for identification of associated markers or candidate genes. Since last 20 years, GWAS/MTA approach has been used in various plant species to understand the mechanism of acclimatization to different stresses and to look about the genetic basis of desired traits during stress induction. In case of legumes, GWAS/MTA studies related to cold stress tolerance are limited. GWAS was conducted in white clover for identification of cold tolerance-related traits. 53 loci associated with cold tolerance traits were identified from 192 diverse germplasm collected from Patagonia region of South America. 17 of the 53 SNP trait relationships regulated several traits and were stable across many sites, according to the study carried by Inostroza and co-workers. This work was the first one to establish a QTL for cold tolerance-related phenotypes, and it revealed its genetic basis as well as prospective genomic areas for future functional validation investigations (Inostroza et al., 2018). In pea plant, 62 SNPs significantly associated with frost tolerance at six different linkage groups were identified by GWAS in set of 365 pea accessions (Beji et al., 2020). A total of 50 candidate genes representing significant SNPs annotation linked to frost damage (FD) loci were detected by GWAS (Beji et al., 2020).

## Transcriptomics: A Link to Have Insight Into Genes Regulating Cold Stress

Transcriptional response of the genome varies under multiple biotic and abiotic stresses in different tissues. Transcriptomic analysis initially relied on the identification of differentially expressed genes. Gene microarray, expressed sequence tag-based method (EST, SAGE), and Next-Generation Sequencing (NGS) based RNA-sequencing technology were developed to obtain the transcriptome as well as identifying differentially expressed genes (Mortazavi et al., 2008; Nagalakshmi et al., 2008; Wilhelm et al., 2008). The rise of NGS technology and availability of genome sequence has made it possible to further study species or previously unidentified species in resequencing and de-novo sequencing aspects (Miao et al., 2015). The technology (RNA-Seq), is making important in-roads to genome annotation by allowing the sequencing of the entire transcriptome and provides expression profiles of either coding or non-coding RNAs (Miao et al., 2015). Many important genes/transcriptional factors (TFs) expressed during developmental, physiological, or pathological mechanisms, biotic and abiotic stresses were identified by these means. Gene expression analysis under cold stress in different plant model systems was reported by many researchers. Many transcriptomic studies in *A. thaliana* have been undertaken in order to decipher cold responses (Bahrman et al., 2019). Some high throughput transcriptomic analyses have been performed in some legumes (Fabaceae), such as *Vicia faba* (Lyu et al., 2021), *Pisum sativum* (Bahrman et al., 2019), *Arachis hypogaea* (Jiang et al., 2020), *Ammopiptanthus mongolicus* (Pang et al., 2013), *Glycine max* (Kidokoro et al., 2015), *Lotus japonicus* (Calzadilla et al., 2016), *Vigna unguiculata*, subspecies sesquipedalis (Tan et al., 2016), *Vigna subterranean* (Bonthala et al., 2016), *Medicago falcate* (Miao et al., 2015), *Medicago sativa* (Song et al., 2016), and *Cicer arietinum* (Sharma and Nayyar, 2014). Transcriptomic studies in these legume species under the exposure of cold stress have led to altered transcript of genes (Buti et al., 2018; Guan et al., 2019). These changes in the transcriptome are regulated by a large number of different TFs and various other key regulatory genes. TFs act as transcriptional inducers or repressors in the regulation of development, metabolic processes, biotic and abiotic stresses (Ng et al., 2018) including cold stress (Jiang et al., 2020). TF family members like APETALA2/ETHYLENE RESPONSIVE FACTOR (AP2/ERF) (Nie et al., 2018), WRKY (Birkenbihl et al., 2017), and basic helix–loop–helix protein (bHLH) are differentially expressed and play a significant role in providing low temperature stress tolerance in plants (Sun et al., 2018). Identification of cold-responsive TFs helped to demonstrate cold resistance and tolerance in legume plants. For instance, one of the family member of AP2/ERF TF, such as C-repeat binding factors/dehydration responsive element binding factor1 proteins (CBFs/DREB1s), are proved to play an important role in plant cold tolerance (Jiang et al., 2020). CBFs in response to cold stress are rapidly over expressed in a short period of time (15 min) in plants (Kidokoro et al., 2015). CBFs are reported to directly regulate the expression levels of cold-responsive (COR) genes, thus can help in enhancing cold tolerance in many legume species (An et al., 2016; Zhang et al., 2018). Although, there is a need to recognize different cis elements present in CBF promoters and combine them with different TFs to regulate the expression of CBFs under cold stress in legume plants. ICE1 (inducer of CBF expression 1) is an MYC-like bHLH transcriptional activator, which induces the expression of CBF genes by binding to the CBFs’ promoters under cold stress in plants (Kim et al., 2015). Detail of some important genes/transporters/transcription factors identified by various scientific groups on expression studies under cold stress in legumes are tabulated in Table 3.

**Table 3:** Genes/transcription factors identified having role in cold tolerance in different legumes by using RNA seq technology.

S.No.	Gene/transcription factors	Regulation	Tissue	Plant	Reference
1	WCOR413-15785	Down	Leaf	*Vicia faba*	Lyu et al., 2021
2	DHN2-12403	Up			
3	DHN2-14197	Up			
4	DHN2-14797	Down			
5	HVA22-15951	Up			
6	COR15-14478	Down			
7	DREBs	Up	Leaf	*Arachis hypogaea*	Wang et al., 2021
8	Phytochrome interacting factors	Up			
9	Raffinose synthases	Up			
10	Galactinol synthase	Up			
11	CBF1	Up	Shoot	*Lotus japonicus*	Calzadilla et al., 2016
12	CBF3	Up			
13	ICE1	Down			
14	RD29A	Up			
15	COR47	Up			
16	NAC	Up	Leaf	*Arachis hypogaea.*	Jiang et al., 2020
17	WRKY	Up			
18	ERF	Up			
19	MYB	Up			
20	C2H2	Up			
21	AP2-EREBP	Up	Leaf	*Pisum sativum*	Bahrman et al., 2019
22	bHLH	Up			
23	AP2-EREBP	Up			
24	Cold, circadian rhythm, RNA-binding 2, GRP7	Up			
25	Cation efflux system protein	Up	Leaf	*Cicer arietinum*	Sharma and Nayyar, 2014
26	L-ascorbate oxidase like protein	Up	Gynoecium		
27	Beta-galactosidase	Up			
28	Sucrose phosphorylase	Up	Anther		
29	Translation initiation factor EIF-2B epsilon	Up			
30	Peroxisomal ABC transporter	Up			
31	Wound responsive protein	Up	Root		
32	zinc finger family (including C2C2, C2H2, and C3H)	Up	Leaf	*Vigna unguiculata*	Tan et al., 2016
33	JUMONJI	Up			
33	Psudo ARR	Up			
34	PHD	Up			
35	ELF3	Up			
36	AtSR	Up			
37	Auxin responsive factor	Up			
38	WHIRLY2	Up	Leaflet	*Vigna subterranea*	Bonthala et al., 2016
39	GATA9	Down			
40	GRAS	Up	Leaf	*Medicago sativa*	Zhuo et al., 2018
41	HSF	Up			
42	FAR1	Up	Leaf	*Medicago falcata*	Miao et al., 2015
43	Orphans	Up			
44	MADS	Up			

## Proteomics: To Study the Change in Proteome Caused by Cold Stress

Proteomics deals with the in-depth study of proteome (gene product present in different tissue or organelle at distinct cell developmental stages), protein–protein interactions, protein accumulation, post-translational modifications (PTMs), and analysis of gene product (Chakraborty et al., 2015; Larance and Lamond, 2015; Minton, 2016). The post-transcriptional modifications like RNA splicing leading to different protein isoforms and the PTMs as processes resulting in multiple functional proteoforms encoded by a single gene make the proteome analysis complex indicating modulation of final protein product of a single gene in response to environmental cue (Kosová et al., 2021) Proteins can act as molecular chaperones, enzymes, and TFs that might play an important role in regulating stress signaling and protecting plants from stress (Rodziewicz et al., 2014). Numerous cold stress-related studies conducted on various plant species revealed that cold stress led to change in proteins response. Different protein families that play a vital role in cold stress response have been identified while studying differential proteomics in different plant species (Xiaoqin et al., 2009; Weckwerth et al., 2015). Several proteomic studies have been carried out in different legumes under various biotic and abiotic stresses (Table 4). However, a few studies related to cold stress have been conducted in legumes like soybean, chickpea, common bean, mung bean, grass pea, and forage legumes (Jan et al., 2022). Some of the cold tolerance proteomic studies in important legume crops are discussed under this section:

**Table 4:** Proteomic studies related to cold stress conducted in various legumes.

S.No.	Plant	Source	Approach used	No. of protein identified	Functions	Reference
1	Soybean (*Glycine Max*)	Root	LC/nanoESI-MS	59	Plant defense, translocation and storage, various metabolic pathways, secondary metabolism, protein synthesis, growth and development, cellular and electron transport	Swigonska and Weidner, 2013
		Seed	2-DE and MALDI-TOF/MS	40	Cell defense, energy, protein synthesis, cell growth/division, storage, transcription and transport.	Cheng et al., 2010
		Leaf	2-DE and MALDI-TOF/TOF MS	57	Transcription and translation regulation; photosynthesis; protein folding and assembly; defense; storage proteins; signal transduction; metabolic pathways (carbohydrate, lipid, energy, amino acid, nitrogen)	Tian et al., 2015
2	Pea (*Pisum sativum*)	Leaf, stem, root	2DE, ElectroSprayIonisation (ESI)	68	Photosynthesis and defense, energy metabolism	Dumont et al., 2011
		Leaf (stromal and luminal chloroplasts proteome)	2DE, 2D-DIGE, MALDI TOF-TOF	620 spots in the stromal pea proteome and 400 spots in the lumenal pea proteome	Soluble sugar synthesis, antioxidant potential, regulation of mRNA transcription and translation	Grimaud et al., 2013
		Leaf(mitochondrial proteome)	2DE, Q-TOF MS	33	Photosynthetic and respiratory rates	Taylor et al., 2005
		Root	2DE, MALDI TOF/TOF	74	Ca ^2+^ dependent signal transduction pathways associated proteins and cell division and expansion	Badowiec et al., 2013
3	Common bean (*Phaseolus vulgaris*)	Root	2DE, MALDI TOF/TOF	64	Protection against stress, cell cycle regulation and hormone synthesis, regulating metabolic pathways	Badowiec and Weidner, 2014
4	Chick pea (*Cicer arietinum*)	Seedling	MALDI-TOF-TOF and LC–MS/MS	70	cellular organelles (mitochondria, chloroplast), protein involved in defense system, metabolic pathways	Heidarvand and Maali-Amiri, 2013
5	Mung bean (*Vigna radiata*)	plumule or epicotyl	2DE, MALDI-quadrupole (Q)-TOF MS/MS and Western blotting	17	cell growth, wall formation, ATP production, the stress response, and methionine assimilation.	Huang et al., 2006
6	Red clover (*Trifolium pratense*)	Roots	2D-DIGE, MALDI TOF-TOF/MS	408	carbohydrate and energy metabolism, amino acid metabolism,signal transduction, molecular chaperones and protein folding, transcription and translation and metabolite transport	Bertrand et al., 2016

Cold stress has affected soybean production by 47–63% (Vara Prasad et al., 2000). Different gel-based and gel-free proteomics methods like 2DE, mass spectrometry, LC–MS, MALDI, iTRAQ have been utilized for analyzing protein change in tissue or organelle proteome under cold stress (Swigonska and Weidner, 2013). The proteome analysis of roots of soybean cv. Aldana under prolonged cold and osmotic stress revealed 59 differentially expressed proteins in control as well as treated samples (Swigonska and Weidner, 2013). LC/nanoESI-MS method was employed for identification of differentially expressed proteins that were involved in different functional categories like plant defense, translocation and storage, various metabolic pathways, secondary metabolism, protein synthesis, growth and development, cellular and electron transport (Swigonska and Weidner, 2013). It was also revealed that 24% of identified proteins were responsible for growth and development followed by 22% for translocation and storage (Swigonska and Weidner, 2013). Another study on seed proteomics of soybean under cold stress suggested that there is an upregulation in expression of the different proteins like glutathione S transferase, sucrose binding protein, and dehydrins while downregulation in expression of proteins responsible for cell division and growth, transcription, protein synthesis and storage metabolism (Cheng et al., 2010). Further analysis of leaf proteome of cv. Guliqing (cold-tolerant) and cv. Nannong 513 (cold-sensitive) soybean cultivar., 57 differentially abundant proteins were identified by MALDI-TOF/MS method (Tian et al., 2015). The identified proteins play an important role in various metabolic pathways and cellular processes, such as transcription and translation regulation, photosynthesis, protein folding and assembly, defense, storage proteins, signal transduction, metabolic pathways (carbohydrate, lipid, energy, amino acid, and nitrogen) (Tian et al., 2015). This study suggested that the presence of more proteins related to lipid and polyamine biosynthesis, photosynthesis, and metabolic recycling whereas less ROS production, low protein proteolysis, and energy depletion is responsible for cold stress tolerance in soybean cv. Guliqing under cold stress. However, these findings could provide an in-depth knowledge on cold stress responses and cold tolerance mechanisms in spring soybean (Tian et al., 2015).

Like other legume crops, pea and common bean crop yield is also affected by cold stress. Proteomics analysis of different pea cultivar revealed the role of various proteins, such as cyclophilin, caffeoyl-CoA O-methyltransferase, plastoglobulin, glycine decarboxylase-H subunits protein disulfide isomerase, and disease resistance protein for providing tolerance in pea under cold stress (Sharma et al., 2005; Dumont et al., 2011). In a study on pea crop, proteomic technique 2DE followed by Electrosprayionization (ESI) was used to have better insight into cold tolerance in two contrasting pea lines, that is, Champagne (resistant) and Terese (sensitive). Three different tissues (leaf, stem, and root) were studied and 68 differentially expressed proteins were identified (Dumont et al., 2011). An increase in the protein expressions responsible for photosynthesis and defense are responsible for more adaptations to cold stress (Dumont et al., 2011). Frost resistance in pea crop might be due to re-orientation of proteins involved in energy metabolism (Dumont et al., 2011). Effect of cold stress on chloroplast of two contrasting pea lines (Champagne and Térèse) was assessed by 2D-DIGE technique suggested that chilling tolerance might be due to change in proteins associated with soluble sugar synthesis, antioxidant potential, regulation of mRNA transcription and translation in chloroplast (Grimaud et al., 2013). An increase in proteins related to carbohydrate, protein synthesis, and photosynthesis was observed in pea plant chloroplast under cold stress (Grimaud et al., 2013). Cold stress leads to alteration in photosynthetic and respiratory rates of pea leaves (Taylor et al., 2005). To understand the impact of environmental stresses like cold, drought, and herbicide on mitochondrial proteome, gel electrophoresis, and MS approach was utilized in pea crop (Taylor et al., 2005). Cold stress significantly affected the leaf metabolism and caused oxidation of mitochondrial proteins without leading to accumulation of lipid peroxidation products inside mitochondria (Taylor et al., 2005). This also suggested that the oxidative stress due to cold and drought conditions is not severe like oxidative stress caused by application of herbicides to pea plant (Taylor et al., 2005). Root growth and development in pea and common bean plant is also affected by both long and short-term cold stress. Root proteome analysis of pea as well as common bean plant under long and short-term cold stress by using 2DE, MALDI-TOF approaches identified proteins involved in regulating metabolic pathways, protection against stress, cell cycle regulation, and hormone synthesis that might have effect on root growth and development in the early stages of plant life (Badowiec et al., 2013; Badowiec and Weidner, 2014). An increase in the Ca^2+^-dependent signal transduction pathways associated proteins and cell division and expansion regulating proteins can be seen in roots of pea and common bean plant affected by cold stress (Badowiec et al., 2013; Badowiec and Weidner, 2014). Response of legume like pea and common bean to cold stress is directly proportional to the length and manner of cold stress exposure, which ultimately alter the root proteome of plants (Badowiec and Weidner, 2014).

In case of chickpea, cold stress declines the crop production by 45–61% (Sharma et al., 2016). MALDI-TOF and LC–MS/MS were used to analyze change in proteome of chickpea seedlings of cv. Sel 96Th11439 at early developmental stage under cold stress (Heidarvand and Maali-Amiri, 2013). Increase in expression of various proteins localized in different cellular organelles (mitochondria, chloroplast), protein involved in defense system, metabolic pathways were found in providing cold stress tolerance to chickpea under prolonged stress (Heidarvand and Maali-Amiri, 2013). A total of 70 differential expressed proteins were identified out of which 4 major proteins involved in providing cold stress tolerance in chickpea are globulin protein, FK506-binding protein (FKBP), NADP^+^ binding Rossmann domain-containing protein, and a protein containing cyclophilin ABH-like region (Heidarvand and Maali-Amiri, 2013). Brassinosteroid (BR) synthetic gene plays an important role in cell elongation, plant growth, development, and responses to several stresses (cold, heat, and drought). The function of BR synthetic gene is suppressed by cold stress in various plants. Applying exogenous BR to epicotyls of mung bean plant helps in regulating epicotyl growth and to overcome inhibitory growth effect by cold stress (Huang et al., 2006).

## Metabolomics: Knowledge of Metabolites Regulating Cold Stress Response

Metabolomics is the scientific study of the metabolites and processes that comprise cellular metabolism. In specific, it is concerned with distinguishing the characteristics of individual cells, and laying down their consequences (Daviss, 2005; Gong et al., 2013). Molecular metabolomics has made it possible to gain greater insights into multiple tolerance mechanisms at metabolic levels under abiotic stress (Bokszczanin and Fragkostefanakis, 2013). Physiological, biochemical, and molecular mechanisms are implemented by plants against a wide variety of stresses, such as biosynthesis of various metabolites, activation of antioxidant enzymes, transport of ions, accumulation of osmoprotectants, and release of different plant hormones (Pandey et al., 2015; Singhal et al., 2021). Furthermore, plants undergo different metabolic changes against stress conditions by synthesizing compatible solutes, antioxidants, and stress-responsive proteins, which act as anti-stress factors (Ramalingam et al., 2015). The ability of plants to withstand low temperature leads to an increase in freezing resistance and tolerance by induction of different mechanisms, such as cold acclimation (Dale and Fortin, 2014). When metabolite profiles are studied at large scale, it is possible to acquire observations of precursors, intermediates, and consequences of metabolic processes. Using it, one can identify and observe undiscovered mass spectral tags (MSTs) as well as establish metabolites, which play significant roles in metabolism, physiology, and stress tolerance (Zhou et al., 2019). Researchers used metabolomics in legumes to study reaction of *Medicago truncatula* to diverse stressors (Bell et al., 2001). In terms of metabolites, such as amino acids, carbohydrates, organic acids, and free fatty acids, both technologies provide complementary perspectives. However, majority of metabolomic platforms still require quality assurance and method validation. In order for metabolomics to improve in the future, we need databases, experimental standards, and data exchangeability between laboratories to be fulfilled (Sansone et al., 2007; Weckwerth, 2011). Even in the midst of the difficulty of conducting comprehensive metabolomic studies, it has been possible to conduct a variety of specific analyses to investigate the role of subsets of metabolites in various conditions, such as cold stress. The potential significance of genome research in crop development is further emphasized by preliminary findings from integrating metabolic techniques with transgenics, which suggest a possibility to boost intrinsic stress resistance in legume crops (He and Dixon, 2000; Wu and VanEtten, 2004). Some of the metabolites studied under cold stress in legumes are enlisted in Table 5. Further, the role of metabolomics during cold stress conditions in different legumes is discussed in following sections:

**Table 5:** Overview of metabolic studies related to cold stress effects on some legumes.

Legume	Tissue	Method	Metabolites studied	Reference(s)
Peanut (*Arachis hypogaea*)	Leaf	GC–MS	Amino acids, sugars, sugar alchols, fatty acids	Patel et al., 2022
Lotus (*Lotus japonicus*)	Shoot	Expression analysis, Illumina	Sucrose, terpenoids, anthrocyanin	Calzadilla et al., 2016
Chickpea (*Cicer arietinum*)	Leaf	GC–MS, qRTPCR	Oxalic acid, polyamines, putrescine, CAT, LOX, SOD	Kazemi Shahandashti et al., 2013; Amini et al., 2021
Common bean (*Phaseolus vulgaris*)	Seed	MS, Hybrid Orbitrap	Flavoinoids, phenol lipids, isoflavoinoids	Mecha et al., 2022
Alfalfa (*Medicago sativa*)	Leaf	Biochemical assays	Raffinose and D-maltose, total soluble amino acids, sugars, and proline	Zhuo et al., 2018; Shi et al., 2019
Soybean (*Glycine max*)	Seedling	GC–MS/ HPLC	Genistein, genistin, daidzein, succinate, pyruvate	Janas et al., 2002; Ramalingam et al., 2015; Yeshi et al., 2021

Alfalfa *(Medicago sativa L.)* is a high feed crop commonly grown in different countries where winters are bitterly cold which pose a barrier to regeneration and growth of alfalfa, resulting in significant economic deficits. Enhancing alfalfa’s capacity to withstand the winter has emerged as a critical production concern. A study was performed by employing biochemical and metabolomic assays to compare the resistance against freezing in alfalfa grown under two different soil water regimes to understand how water shortage impacts tolerance against freezing stress. Water-stressed alfalfa had lower semi-lethal temperatures than the well-watered alfalfa. Under water scarce and low-temperature circumstances, the pool proportions of total soluble amino acids, sugars, and proline altered significantly. Metabolomic findings indicated 72 distinct subclasses of differential metabolites *viz* lipid and lipid-like molecules (e.g., glycerophospholipids, fatty acids, and unsaturated fatty acids) and peptides, amino acids, and analogues (e.g., proline betaine), were highly expressed in water scarce conditions. Certain flavonoids and carbohydrates, like raffinose and D-maltose, were elevated at low temperatures. Investigations of the Kyoto Encyclopedia of Genes and Genomes indicated the existence of 18 highly enriched pathways implicated in the production and metabolism of amino acids, carbohydrates, glycerophospholipids, and unsaturated fatty acids (Shi et al., 2019). Another study carried by Zhou and co-workers where alfalfa was subjected to cold stress at 4°C for different time lines. The high-throughput sequencing identified 50,809 annotated unigenes and 5,283 genes that were differentially expressed (DEGs). According to metabolic pathway enrichment analysis, DEGs were shown to be implicated in plant hormone signal transduction, glucose metabolism, photosynthetic signal transduction, and synthesis of amino acids. The peroxidase and catalase activity were also found to fluctuate in a manner that was in line with changes in the gene transcript profiles that were being studied (Zhuo et al., 2018).

**Figure 3:**
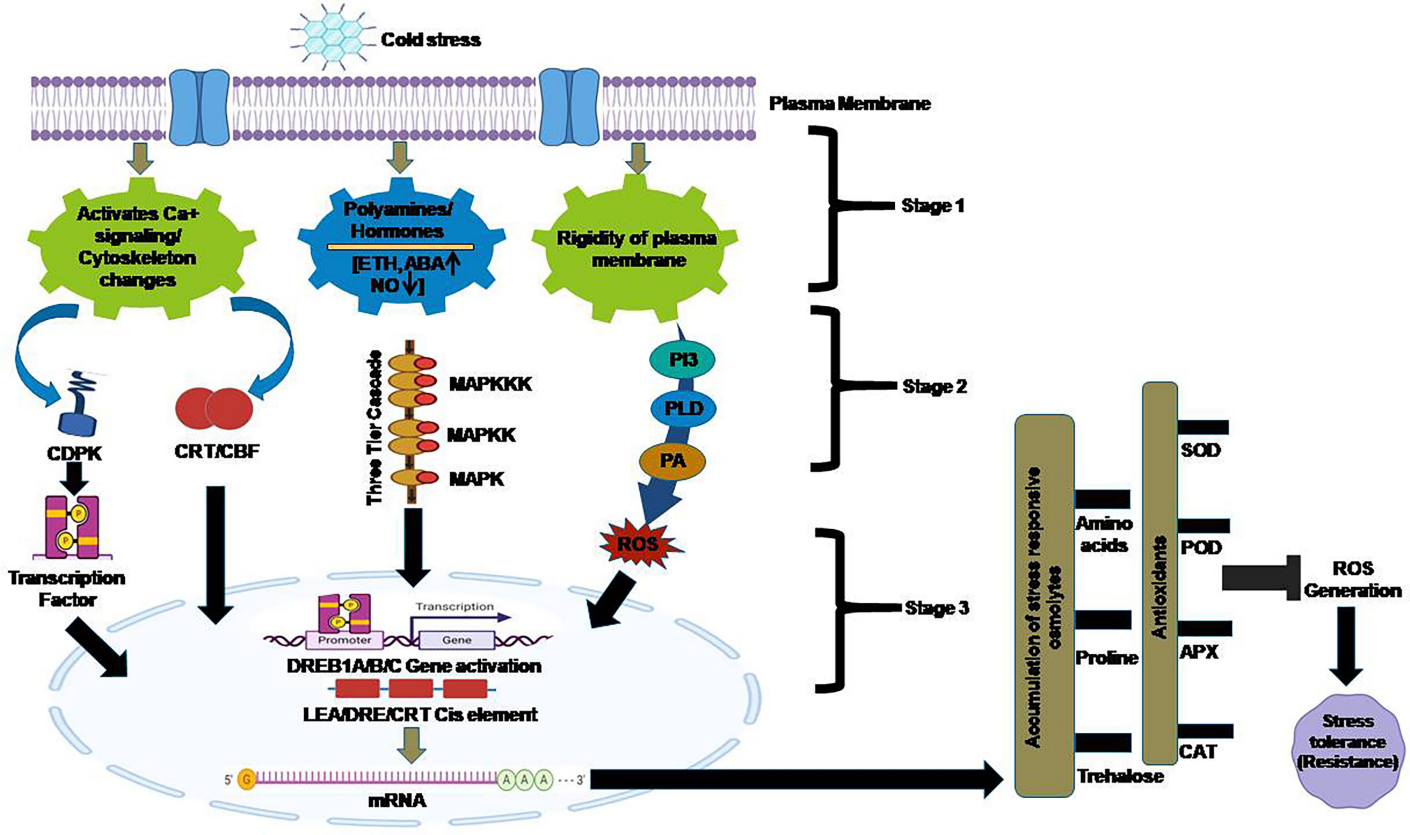
Cold stress-induced signal transduction and response: Cold stress is perceived at the plasma membrane with activation of downstream signaling cascade *viz* activation of calcium signaling, polyamines and hormone signaling and rigidity of plasma membrane which in turn activates multiple cytoplasmic proteins and expression of different genes which ultimately leads to cold tolerance. CDPK: Calcium-dependent Kinases; CBF: C repeat binding factors; MAPK: Mitogen-activated protein kinase; ROS: Reactive oxygen species.

Chickpea is the second most essential edible crop in the world (FAOSTAT, 2021). Chickpea is one of the important sources of easily digestible protein and minerals. As a key method to reduce yield loss during typical spring sowing in regions with hot and dry weather, early planting of chickpea in autumn or spring is preferred in Mediterranean regions. A key limiting factor for early chickpea seeding is, however cold stress sensitivity (Kazemi-Shahandashti et al., 2014). When a stress signal is sensed by receptors, a variety of components of the signaling pathway get involved in signal transduction, including cell membrane lipids, reactive oxygen species (ROS), calcium, hormones, protein kinases, and phosphatases (Kazemi-Shahandashti and Maali-Amiri, 2018). Plants adapt to cold stress by changing transcription factors, reprogramming their gene expression in response to stress, and altering their metabolism (Novák et al., 2016) as cold stress is perceived at the plasma membrane with activation of downstream signaling cascade (Figure 3). Chickpeas grow poorly at low temperatures since sporogenesis, pollen germination, pod abortions, and blooming is impaired (Kumar et al., 2010). The decline in enzyme activity and reaction rates associated with temperature is not only due to a decrease in enzyme activity and reaction rates (Levitt, 1980), but also because of active metabolic re-organization (Cook et al., 2004; Kaplan et al., 2004). When cold stress is applied to immature microspores, transcription of a particular gene OCINV4 (tapetum specific invertase gene) is inhibited thus preventing sugars from reaching the tapetum and pollen grains and allowing another type of sucrose to accumulate (Oliver et al., 2005). Affected by sterility of the pollen, the entire reproductive process is affected and polyamine levels fluctuate in response to cold stress and then fall over the course of prolonged exposure (Nayyar et al., 2005). This polyamine putrescine increases the weight of viable pods and seeds which in turn enhances tolerance of plants to cold stress. Defensive and mitochondrial responses among the sensitive (ILC533) and tolerant (Sel96Th11439) genotypes of chickpea which are responsible for cold stress tolerance were identified in a study (Karami-Moalem et al., 2018). Cold stress responses are translated into different physiological changes as a consequence of direct or indirect gene expression and are regulated by factors, such as DNA methylation. During cold stress tolerance, there is significant increase in the antioxidant enzymatic activities, such as catalases (CAT), superoxide dismutase (SOD), and ascorbate peroxidase (APX), along with the increase in non-enzymatic molecules, such as proline and ascorbate, to eliminate the effects of oxidative stress in chickpea thus, validating the active scavenging system against ROS (Karami-Moalem et al., 2018). Chickpea tolerant cells in response to cold stress modify their genome, such as the number of bands, was found to be decreased in tolerant genotypes. During Cold stress, the methylation levels are found higher in comparison to de-methylation (27.92 *vs* 22.09%) in susceptible ones and (29.05 vs. 19.79%) in tolerant genotypes (Rakei et al., 2016). The approaches for cold stress analysis, such as physiological and biochemical analysis, established the involvement of lipoxygenases (LOX), antioxidants, fatty acid content as cell responses during cold stress. The alterations in membrane fatty acid compositions, defense machinery (such as anti-oxidative enzymes), and damage indicators, including the malondialdehyde (MDA) and electrolyte leakage index (ELI) in chickpeas were found throughout the cold acclimation (CA), cold stress and recovery (R) phases. According to these findings, the proportion of unsaturated to saturated fatty acids increased, which is a mark of cold resistance, notably after the CA phase. Antioxidant enzymes were influential throughout the CA and R stages, but cold stress decreased their activity, suggesting that other metabolites or enzymes are involved in plant cold tolerance development. The expression pattern of certain enzymes like CAT, LOX, and SOD, was investigated during experimental treatments employing quantitative real-time PCR. The LOX activity exhibited a bidirectional association with membrane damage index in CA and an exciting link with double bond index in cold stress, implying a role in secondary metabolite signaling pathways, such as jasmonic acid signaling (Kazemi Shahandashti et al., 2013).

Soybean, an important food crop worldwide is a valuable source of proteins consumed globally, (Islam et al., 2019). As an additional method of improving soil and its fertility, soybeans utilize natural nitrogen fixation in the roots. However, a number of abiotic factors, such as cold, heat, drought, and high salt concentration hinder soybean growth and productivity (Hasanuzzaman et al., 2016). There are a number of defense mechanisms activated by complex transcriptional regulatory networks at the physiological and biochemical level, including activation of chaperones, detoxifying enzymes, transporters, and enzymes for metabolite production (Pandey et al., 2021). A study was carried out using 5 concentrations of 5-aminolevulinic acid (ALA) added to the Hoagland solution for 12 h for the purpose of increasing soybean plant cold stress resistance. The plants treated with ALA were exposed to cold stress at 4°C for 48 h after treatment. On average, ALA at low concentrations (5–10 uM) increased relative water content (RWC) and chlorophyll content compared to non-ALA-treated plants. The amount of reactive species generated by thiobarbituric acid (TBARS) in plants pre-treated with ALA (15–40 uM) increased dose dependently with about 117% in plants treated with 40 uM. Furthermore, 5 uM ALA pre-treatment resulted in maximum cold tolerance (Balestrasse et al., 2010). This suggests that heme oxygenase (HO-1) is an antioxidant as well as a rate-limiting enzyme in the heme catabolism. By increasing the activity of heme proteins, such as CAT and promoting heme catabolism, ALA, an endogenous plant growth regulator, is an effective treatment for combating cold stress in soybean. As a consequence, the highly antioxidant substance biliverdin is produced, along with carbon monoxide without affecting growth (Balestrasse et al., 2010). A study carried out by Xu and co-workers on different temperature conditions of soybean was carried out where a controlled environment with low (22/18°C), optimal (28/24°C), and high (36/32°C) temperatures was used to assess the cumulative influence of temperature and CO_2_ on leaf metabolites, photosynthetic activities, and growth. Through the range of CO_2_ levels studied, the photosynthesis rate, mesophyll, and stomatal conductance, photosystem II quantum yield, and electron transport increased. The photosynthetic restriction was temperature-dependent and affected by photo-biochemical and metabolic processes. Under well-watered and nutrient-sufficient circumstances, CO_2_ tended to fully or partially compensate for the respective low temperature and high temperature stressors (Xu et al., 2016).

In semi-arid tropical and subtropical regions, peanut forms an important grain legume that is farmed for its edible oil content and proteins (Zhang et al., 2019). Temperature is an important factor in the development and growth of this crop, as it demands a high range of temperature throughout the entire process (Wang et al., 2003). It is known that peanuts germinate at 12–15°C, and reach their optimum growth at 28°C, but these plants suffer drastic metabolic disturbances below 12°C, a kind of stress condition (Bell et al., 1994). All of these factors influence chilling tolerance that includes their structure, composition, and metabolism. One of the primary ways in which plants adapt to temperature changes is through the distribution ratio of lipids on their membranes and un-saturation of the glycerol lipid group. Peanut plasma membranes are less elastic, and membrane lipids are reduced to a gel-like structure under cold stress (Murata et al., 1982), which results in poor protoplast flow and increased membrane permeability, causing electrolyte leakage and an imbalance of intracellular ions (Huang et al., 2015). A group of cold-responsive metabolites was discovered during the metabolomic study of two peanut cultivars exposed to chilling stress, including various sugars and polyamines. Under cold stress, these compounds accumulated more in the cold-resistant variety (SLH) than in the cold-susceptible variety (ZH12), demonstrating their role in protecting peanuts against chilling damage (Wang et al., 2021). Cold-tolerant plants exhibit greater growth amplitude of these osmotic regulatory molecules than cold-sensitive plants due to their increased levels of proline, soluble carbohydrates, and soluble proteins in the cytosol. During extremely cold temperatures, these compounds are drastically reduced in the cytosol of the plant (Bai et al., 2018; Kazemi-Shahandashti and Maali-Amiri, 2018). Considering that most metabolic pathways are connected in intricate systems, modifying one metabolic pathway may have adverse consequences on another, which may lead to undermining the manipulated crop. A large-scale metabolic experiment is required in order to observe the metabolic networks that have a significant role in the development and growth of legumes.

## Trans-Genomics: Use of Gene-Based Approach to Understand Cold Tolerance

Traditionally, crop development for cold tolerance by a standard breeding approach has been limited due to stress-tolerant trait complexity, absence of precise phenotyping methods, selection criteria, and little genetic diversity in the respective breeding populations (Sanghera et al., 2011). Advances in recombinant DNA technology and development of effective genetic engineering methods can aid in the development of precise methodology and strategies for producing cold-tolerant cultivars in a variety of agricultural species (Wani et al., 2016). Transgenic technology or simply transgenomics by the use of gene-based approaches may prove valuable for gaining deep insight into stress tolerance mechanisms and, as a complementary means, for genetically improving crops, which in turn can help to alleviate some of the major pitfalls to crop improvement (Sharma and Ortiz, 2000). A deep study has been carried out for identifying different genes which play an important role in enhancing cold or freezing tolerance in plants. The central principle for transformation experiments revolves around enzymes important for biosynthesis of various osmoprotectants, detoxifying enzymes, late embryogenesis abundant (LEA) proteins and those which encode for membrane lipids (Eapen, 2008). Many aspects of cold adaptation are under transcriptional control which helps in choosing regulatory factors for introgression of these genes that are believed to be responsible during putative tolerance and stress response, thus paving a way for developing varieties with improved cold tolerance. There have been reports of genetic transformation of all important legume crops, including *Vigna species, Chrysanthemum arietinum, Chrysanthemum cajan, Phaseolus* species, *Lupinus* species, *Vicia* species, and soybeans. With the exception of soybean, transgenic grain legumes, unlike their cereal counterparts, are yet to migrate out of laboratories to major farm fields, regardless of their importance to tropical agriculture (Eapen, 2008). Transgenic alfalfa’s increased tolerance to aluminum toxicity (Tesfaye et al., 2001) and soybean’s increased tolerance to cyanamide toxicity (Zhang et al., 2005) show this method can be applicable to legumes also. At International Crops Research Institute for the Semi-Arid Tropics (ICRISAT), training on efficient transformation techniques has been provided for legume crops, such as peanuts, pigeon peas, and chick peas. Transgenes are being used to overcome abiotic stressors in various leguminous crops and thus different transgenic methods are developed to improve stress resistance, including those that encode enzymes responsible for biosynthesis of osmoprotectants (Sharma and Lavanya, 2002) or that modify membrane lipids (Sinha et al., 2015), or LEA proteins (Wang et al., 2019), and detoxification enzymes (Kazemi-Shahandashti and Maali-Amiri, 2018). Both Agrobacterium-mediated transformations (Joshi et al., 2018) and particle bombardment methods were utilized in transformation experiments in soybean (Pareddy et al., 2020). Commercially, it is the only legume-based transgenic crop which is currently been grown. Several great reviews on gene technology uses in soybean have been published from time to time to understand the stress biology of the crop (Debbarma et al., 2019). The transformation experiment for abiotic stress tolerance on transgenic soybean where harmful cyanamide was converted to urea, from the soil fungus *Myrothecium* using cyanamide hydratase (Cah) coding sequence has been reported in recent. Cah expression reduced cyanamide levels in soybean leaf callus and embryogenic cultures as evidenced by cyanamide resistance (Zhang et al., 2005). Another study found that transgenic soybeans with constitutive expression of the nectarin 1 (ntr1) gene from *Brassica campestris* accumulated more methyl jasmonate (MeJA). The NTR1 gene codes a plant regulator called jasmonic acid carboxyl methyl transferase that regulates the expression of various plant defense genes against diverse challenges, such as wounding, dehydration, and infections. The transgenic soybean plants’ greater levels of MeJA provided dehydration tolerance throughout seed germination and seedling growth, as measured by the percentage of fresh weight of seedlings.

Significant efforts have been made in soybean to improve and optimize the plant’s cold tolerance. Soybean plants reprogram the expression of many cold-responsive genes against cold stress. Nevertheless, the inherent mechanism underpinning soybean’s cold-stress tolerance is unknown. Research revealed a soybean homolog of the AtTCF1 gene (identified as GmTCF1a), which is involved in tolerance of plant to low temperatures. Cold stress induces GmTCF1a robustly and precisely. Remarkably, higher expression of GmTCF1a ectopically in *Arabidopsis* significantly boosted plant survival. GmTCF1a was mainly induced in response to cold stressors, and ectopic expression of GmTCF1a increased cold resistance and levels of COR15a. These findings suggest that GmTCF1a positively influences cold resistance in soybean, which may bring unique insights into cold-tolerant genetic improvements in crops (Dong et al., 2021). A DREB orthologue, GmDREB3, was identified from soybean employing the RACE technique. Northern blot study revealed that GmDREB3 expression was elevated in soybean seedlings post 0.5 h of cold stress but was not found after 3 h. However, it was not generated by excessive salt or drought stress, nor was it triggered by abscisic acid (ABA) therapy. This reaction was comparable to members of the A-1 subgroup but distinct from other members of the A-5 subgroup, indicating that GmDREB3 gene was engaged in a cold stress-responsive signaling pathway that is independent of ABA (Chen et al., 2009).

MtCTLK1, a cold-tolerant LRR-RLK gene, was discovered in *Medicago truncatula* using transgenic lines overexpressing MtCTLK1 (MtCTLK1-OE). MtCTLK1-OE lines had greater cold resistance than the wild type, but MtCTLK1 lines exhibited lower cold resistance. The decreased tolerance against cold in MtCTLK1 might be compensated for the transgenic expression of MtCTLK1 or its *Medicago falcata* homolog MfCTLK1. Proline buildup, antioxidant enzyme activity, and transcript levels of related genes were enhanced in response to cold, with greater levels in MtCTLK1-OE lines or reduced levels in mtctlk1 lines relative to wild type (Geng et al., 2021). Another investigation found MfAIR12 in *Medicago falcata,* legume germplasm with exceptional cold resistance. MfAIR12 and its *Medicago truncatula* homolog MtAIR12 transcript levels were triggered at low temperature. MfAIR12 overexpression resulted in water buildup in the apoplast with enhancement in cold resistance, which was prevented by water scavengers, demonstrating that the improved tolerance to cold was reliant on the accumulated water. Additionally, a decrease in cold tolerance in *Arabidopsis* mutant air12 was detected, which was recovered by overexpression of MfAIR12. In comparison to wild type, MfAIR12 transgenic lines had elevated levels of ascorbate redox state, and ascorbic acid and transcripts of the CBF transcription factors and their downstream cold-responsive genes, but decreased levels in air12 mutant lines (Wang et al., 2021). Also, MfERF1, a cold-responsive ERF, was identified from *Medicago falcata,* with exceptional cold tolerance. MfERF1 overexpression boosted the tolerance of transgenic tobacco plants to chilling and freezing, but downregulation of the ortholog of MfERF1 in *Medicago truncatula* led to decreased freezing resistance in RNAi plants (Zhuo et al., 2018). Another cold-responsive PIP2 was derived from *Medicago falcate,* with remarkable cold resistance, and the tolerance of transgenic tobacco plants overexpressing MfPIP2-7 to different stressors was evaluated, including nitrate reduction, chilling, and freezing. Four to twelve hours of cold therapy and two hours of ABA treatment activated the MfPIP2-7 transcript. Pretreatment with ABA inhibitor production suppressed cold-induced transcription of MfPIP2-7 in *M falcata,* demonstrating that ABA was engaged in cold-induced transcription of MfPIP2-7 whose overexpression increased the tolerance of transgenic tobacco plants to chilling, freezing, and NO_3_ deficiency compared to the wild type. Additionally, it was found that MfPIP2-7 facilitates water transport in yeast (Zhuo et al., 2016).

Chickpea photosynthetic machinery, for example, can survive oxidative stress by expressing a prokaryotic homolog of choline oxidase (codA) which was proved by an experiment on transgenic chickpea chloroplasts to determine the survival rate during photo inhibitory damage, and it was found that wild-type plants exposed to high light intensity lost a greater level of PS II activity than transgenic chickpea chloroplasts under the same conditions (Sharmila et al., 2009). Researchers found H_2_O_2_ produced during glycine betaine synthesis in the chloroplasts of transgenic chickpea plants is responsible for the development of a stronger antioxidant system (Sharmila et al., 2009). It is important to mention that the P5CSF129A gene, encoding a mutant D1-pyrroline-5-carboxylate synthetase (P5CS) promoting proline overproduction in chickpeas, was introduced in field trials at ICRISAT (Bhatnagar-Mathur et al., 2009). Reduction in MDA levels, as measured by the formation of proline under drought and cold stress, was associated with enhanced levels of proline synthesis and formation in the leaves (Ejaz et al., 2020). During the progressive drying phase, only very few occurrences showed a noticeable increase in biomass, suggesting that over-expression of proline had no useful effect on accumulation of biomass. Over-expression of the P5CSF129A gene only slightly increased transpiration efficiency (TE), indicating that proline was not able to affect the components of yield architecture which are important in reducing the detrimental effects of stress in chickpea (Bhatnagar-Mathur et al., 2009). The effects of cold stress on growth indices and antioxidant responses in seedlings subjected to cold (4°C) stress for 5 days were studied by Oktem and co-workers. They found that the length and fresh weight of shoots fell dramatically, in contrast to an increase in both growth parameters for roots under the same conditions. Under cold stress, the increase in proline levels in shoots and roots was more pronounced (Öktem et al., 2008). These results, like those in other crops, were in line with prior findings. Genes that affect a single protein tend to be less effective when it comes to handling cold stress. Targeting transcription factors that govern the expression of many genes linked with abiotic stress is one way to address the multigenicity of the agricultural plant response to stress. Researchers previously showed that transcription factors that regulate the coordinated expression of multiple stress-related genes in heterologous transgenic plants under laboratory conditions improved stress tolerance in these plants. Hence, a significant number of transgenic chickpeas containing *Arabidopsis thaliana* DREB1A transcription factors, driven by *A. thaliana* RD29A gene are being generated (Khan et al., 2018). To date, individual genes had a limited impact on stress tolerance in plants. However, the simultaneous activation of a subset of those genes by transcription factors is a promising technique (Srivastava and Sahoo, 2021). It has been shown that transgenic plants containing AP2/EREBP genes (DREB1A) can enhance abiotic stress tolerance in crop plants (Singh et al., 2012). Under a constitutive promoter, over-expression of DREB1A proved to be harmful under normal conditions, whereas it was beneficial when a stress was applied. DREB1A expression was reduced in peanuts by using the stress inducible promoter from rd29A instead of CaMV35S (Puli et al., 2021). Peanut genotypes with high transpiration efficiency (TE) and stomatal conductance have largely failed to develop due to the difficulty of solving the stress tolerance problem. Under greenhouse conditions, plants containing *A. thaliana* DREB1A transcription factor, driven by the rd29A promoter, have been found to confer improved cold stress resistance (Gantait et al., 2020). Several transgenic events with diverse responses were chosen for more comprehensive studies on leaves for gas exchange characteristics. To better understand the mechanism behind transgenic environment stress tolerance, the biochemical reactions have been studied under identical cold stress conditions (Khan et al., 2018).

## Conclusion and Future Perspective

Global climate change has resulted in the emergence of complex stress combinations and their effects on crop yield and overall growth in modern day agriculture. Climate change is a multifaceted issue with long-term consequences in the form of many abiotic pressures. Cold tolerance is among one of those abiotic stresses with a complicated feature that results from multiple molecular interactions in an organism’s genome, transcriptome, proteome, and metabolome. Cold tolerance is stage-specific and appears as a response to cell stability toward external stimuli. Despite the fact that genomic loci influencing cold stress tolerance in plants share a degree of homology across species, the relative expression and location of protein/gene products varies with systems between species, and the context defining cold response may differ entirely. Moreover, the function of “omics” technologies in understanding cold stress tolerance in plant species is of enormous importance and immediate solicitation in elucidating new pathways behind such processes. Plants mainly undergo morphological, biochemical, and molecular changes as a result of cold stress. Although most legumes can withstand these fluctuations at low temperature but extended exposure could cause partial or complete failure of the final product. Multiple changes occur throughout the crop life cycle, from germination to harvesting, as a result of such an environment/climate. Crop plants also show a variety of biochemical and molecular manifestations under cold stress. Cold acclimation in response to the freezing stress has been studied extensively in various plants to understand the molecular and physiological status. Contrary to this a little is known about the responses in legumes for the process of deacclimation and reacclimation. Low temperature alters the gene expression of some important gene products which include the dehydrin, Cor, and CBF genes. Legumes are nutritionally as well as economically important crop species and to understand the process of acclimation during freezing or cold stress a lot of research needs to be carried out. Environmental and genotypic impacts, as well as their interactions, end up making elucidating generic responses difficult. When designing studies and evaluating acclimation, deacclimation and reacclimation reactions affecting cold stress tolerance, plant developmental stages, tissue type, and other characteristics, such as light conditions, must be taken into account. Finally, the function of “omics” technologies in understanding cold stress resistance in plant species is of enormous importance with direct solicitation in drawing out new pathways behind such a mechanism and deciphering the genes providing cold tolerance will help to solve the problem of growing seasons of the legumes and help in growing legumes in different seasons of the year. There is a greater need to investigate and concentrate on the genetic features of legumes that enables to resist cold stress effects and continue to grow and develop normally. It was improved even more by finding a wide range of genetic features and mapping them using various gene mapping approaches like QTL mapping and genome-wide association studies. Apart from that, several other osmolytes (such as glycine betaine) and plant hormones (such as SA, ABA, brassinosteroids, and strigolactone) are currently overlooked and can be more promising at regulating plant responses to cold stress using multi-omic approaches. As a result, adopting integrated multidisciplinary methodologies to investigate these gaps and open up new research vistas is urgently required.

## Author Contributions

KAB, RM, MMP, and UU collected the literature and prepared the first draft. KAB and ZB helped in preparing figures. AAS, AA, and PAS helped in editing the manuscript. BB helped in revising the manuscript. AM finalized figures, tables and helped in revising the manuscript. SMZ conceive the idea, prepared the outline, and helped in finalizing manuscript and its revision. All authors contributed to the article and approved the submitted version.

## Funding

RM and SMZ acknowledge the support of funding agency DST KIRAN WOS-B, New Delhi, India (Vide Project Sanction Order No. DST/WOS-B/2018/832). SMZ acknowledges the support of funding agency NMHS GBPNIHESD, Almora, Uttrakhand, India (Vide Project Sanction Order No. GBPNI/NMHS17-18/SG24/622). AM was funded by the Italian Ministry for University and Research’s “Progetti di Ricerca di Rilevante Interesse Nazionale” (PRIN; grant no. 2020HB9PR9). SMZ and AM acknowledge European Union for funding Erasmus Programme (2018–2020 and 2020–2023) that helped in interaction among the authors for preparing this manuscript.

## Conflict of Interest

The authors declare that the research was conducted in the absence of any commercial or financial relationships that could be construed as a potential conflict of interest.

## Publisher’s Note

All claims expressed in this article are solely those of the authors and do not necessarily represent those of their affiliated organizations, or those of the publisher, the editors and the reviewers. Any product that may be evaluated in this article, or claim that may be made by its manufacturer, is not guaranteed or endorsed by the publisher.

## References

[ref001] Adhikari L. Makaju S. O. Lindstrom O. M. (2021). Mapping freezing tolerance QTL in alfalfa: based on indoor phenotyping. BMC Plant Biol. 21:403. doi: 10.1186/s12870-021-03182-4, PMID: 34488630 PMC8419964

[ref137] Amini S. Maali-Amiri R. Kazemi-Shahandashti S. S. López-Gómez M. Sadeghzadeh B. Sobhani-Najafabadi A.. (2021). Effect of cold stress on polyamine metabolism and antioxidant responses in chickpea. J Plant Physiol. 258–259:153387. doi: 10.1016/j.jplph.2021.153387, PMID: 33636556

[ref1] An D. Ma Q. Yan W. Zhou W. Liu G. Zhang P. (2016). Divergent regulation of CBF regulon on cold tolerance and plant phenotype in cassava overexpressing arabidopsis CBF3 gene. Front. Plant Sci. 7:1866. doi: 10.3389/fpls.2016.01866, PMID: 27999588 PMC5138201

[ref2] Badowiec A. Swigonska S. Weidner S. (2013). Changes in the protein patterns in pea (Pisum sativum L.) roots under the influence of long- and short-term chilling stress and post-stress recovery. Plant Physiol. Biochem. 71, 315–324. doi: 10.1016/j.plaphy.2013.08.001, PMID: 24012770

[ref3] Badowiec A. Weidner S. (2014). Proteomic changes in the roots of germinating Phaseolus vulgaris seeds in response to chilling stress and post-stress recovery. J. Plant Physiol. 171, 389–398. doi: 10.1016/j.jplph.2013.10.020, PMID: 24594390

[ref4] Bahrman N. Hascoët E. Jaminon O. Dépta F. Hû J. F. Bouchez O.. (2019). Identification of genes differentially expressed in response to cold in Pisum Sativum using RNA sequencing analyses. Plants 8:288. doi: 10.3390/plants8080288, PMID: 31443248 PMC6724123

[ref5] Bai D.-M. Xue Y.-Y. Zhao J.-J. Huang L. Tian Y.-X. Quan B.-Q.. (2018). Identification of cold-tolerance during germination stage and genetic diver-sity of SSR Markers in peanut landraces of Shanxi Province. Acta Agron. Sin. 44:1459. doi: 10.3724/sp.j.1006.2018.01459

[ref6] Balestrasse K. B. Tomaro M. L. Batlle A. Noriega G. O. (2010). The role of 5-aminolevulinic acid in the response to cold stress in soybean plants. Phytochemistry 71, 2038–2045. doi: 10.1016/j.phytochem.2010.07.012, PMID: 21051062

[ref7] Beji S. Fontaine V. Devaux R. Thomas M. Negro S. S. Bahrman N.. (2020). Genome-wide association study identifies favorable SNP alleles and candidate genes for frost tolerance in pea. BMC Genomics 21, 1–21. doi: 10.1186/S12864-020-06928-W/FIGURES/4 PMC7430820 32753054

[ref8] Bell C. J. Dixon R. A. Farmer A. D. Flores R. Inman J. Gonzales R. A.. (2001). The Medicago Genome Initiative: a model legume database. Nucleic Acids Res. 29, 114–117. doi: 10.1093/nar/29.1.114, PMID: 11125064 PMC29836

[ref9] Bell M. J. Gillespie T. J. Roy R. C. Michaels T. E. Tollenaar M. (1994). Peanut leaf photosynthetic activity in cool field environments. Crop Sci. 34, 1023–1029. doi: 10.2135/cropsci1994.0011183X003400040035x

[ref002] Bertrand A. Bipfubusa M. Castonguay Y. (2016). A proteome analysis of freezing tolerance in red clover (Trifolium pratense L.). BMC Plant Biol. 16:65. doi: 10.1186/s12870-016-0751-2, PMID: 26965047 PMC4787020

[ref10] Bhatnagar-Mathur P. Vadez V. Jyostna Devi M. Lavanya M. Vani G. Sharma K. K. (2009). Genetic engineering of chickpea (Cicer arietinum L.) with the P5CSF129A gene for osmoregulation with implications on drought tolerance. Mol. Breed. 23, 591–606. doi: 10.1007/s11032-009-9258-y

[ref11] Birkenbihl R. P. Kracher B. Somssich I. E. (2017). Induced genome-wide binding of three arabidopsis WRKY transcription factors during early MAMP-triggered immunity. Plant Cell 29, 20–38. doi: 10.1105/tpc.16.00681, PMID: 28011690 PMC5304350

[ref12] Bokszczanin K. L. Fragkostefanakis S. (2013). Perspectives on deciphering mechanisms underlying plant heat stress response and thermotolerance. Front. Plant Sci. 4:315. doi: 10.3389/fpls.2013.00315, PMID: 23986766 PMC3750488

[ref13] Bonthala V. S. Mayes K. Moreton J. Blythe M. Wright V. May S. T.. (2016). Identification of gene modules associated with low temperatures response in bambara groundnut by network-based analysis. PLoS One 11:e0148771. doi: 10.1371/journal.pone.0148771, PMID: 26859686 PMC4747569

[ref14] Buti M. Pasquariello M. Ronga D. Milc J. A. Pecchioni N. Ho V. T.. (2018). Transcriptome profiling of short-term response to chilling stress in tolerant and sensitive Oryza sativa ssp. Japonica seedlings. Funct. Integr. Genomics 18, 627–644. doi: 10.1007/s10142-018-0615-y, PMID: 29876699

[ref15] Calzadilla P. I. Maiale S. J. Ruiz O. A. Escaray F. J. (2016). Transcriptome response mediated by cold stress in lotus japonicus. Front. Plant Sci. 7:374. doi: 10.3389/fpls.2016.00374, PMID: 27066029 PMC4811897

[ref16] Câmara C. R. S. Urrea C. A. Schlegel V. (2013). Pinto beans (Phaseolus vulgaris l.) as a functional food: implications on human health. Agric. 3, 90–111. doi: 10.3390/agriculture3010090

[ref17] Chakraborty S. Salekdeh G. H. Yang P. Woo S. H. Chin C. F. Gehring C.. (2015). Proteomics of important food crops in the Asia Oceania Region: current status and future perspectives. J. Proteome Res. 14, 2723–2744. doi: 10.1021/acs.jproteome.5b00211, PMID: 26035454

[ref009] Chang Y. Liu H. Liu M. Liao X. Sahu S. K. Fu Y.. (2018). The draft genomes of five agriculturally important African orphan crops. GigaScience 8:giy152. doi: 10.1093/gigascience/giy152, PMID: 30535374 PMC6405277

[ref18] Chen M. Xu Z. Xia L. Li L. Cheng X. Dong J.. (2009). Cold-induced modulation and functional analyses of the DRE-binding transcription factor gene, GmDREB3, in soybean (Glycine max L.). J. Exp. Bot. 60, 121–135. doi: 10.1093/jxb/ern269, PMID: 18988621 PMC3071762

[ref19] Cheng L. Gao X. Li S. Shi M. Javeed H. Jing X.. (2010). Proteomic analysis of soybean [Glycine max (L.) Meer.] seeds during imbibition at chilling temperature. Mol. Breed. 26, 1–17. doi: 10.1007/s11032-009-9371-y

[ref20] Cook D. Fowler S. Fiehn O. Thomashow M. F. (2004). A prominent role for the CBF cold response pathway in configuring the low-temperature metabolome of Arabidopsis. Proc. Natl. Acad. Sci. U. S. A. 101, 15243–15248. doi: 10.1073/pnas.0406069101, PMID: 15383661 PMC524070

[ref21] Dale M. R. T. Fortin M. J. (2014). Spatial analysis: a guide for ecologists, second edition, Cambridge University Press, Cambridge.

[ref22] Daviss B. (2005). Growing pains for metabolomics: the newest’omic science is producing results--and more data than researchers know what to do with. Sci. 19, 25–29.

[ref23] Debbarma J. Sarki Y. N. Saikia B. Boruah H. P. D. Singha D. L. Chikkaputtaiah C. (2019). Ethylene response factor (ERF) family proteins in abiotic stresses and CRISPR–Cas9 genome editing of ERFs for multiple abiotic stress tolerance in crop plants: a review. Mol. Biotechnol. 61, 153–172. doi: 10.1007/s12033-018-0144-x, PMID: 30600447

[ref24] Dong Z. Wang H. Li X. Ji H. (2021). Enhancement of plant cold tolerance by soybean RCC1 family gene GmTCF1a. BMC Plant Biol. 21:369. doi: 10.1186/s12870-021-03157-5, PMID: 34384381 PMC8359048

[ref25] Dumont E. Bahrman N. Goulas E. Valot B. Sellier H. Hilbert J. L.. (2011). A proteomic approach to decipher chilling response from cold acclimation in pea (Pisum sativum L.). Plant Sci. 180, 86–98. doi: 10.1016/j.plantsci.2010.09.006, PMID: 21421351

[ref26] Dumont E. Fontaine V. Vuylsteker C. Sellier H. Bodèle S. Voedts N.. (2009). Association of sugar content QTL and PQL with physiological traits relevant to frost damage resistance in pea under field and controlled conditions. Theor. Appl. Genet. 118, 1561–1571. doi: 10.1007/s00122-009-1004-7, PMID: 19322559

[ref27] Eapen S. (2008). Advances in development of transgenic pulse crops. Biotechnol. Adv. 26, 162–168. doi: 10.1016/j.biotechadv.2007.11.001, PMID: 18055156

[ref28] Ejaz S. Fahad S. Anjum M. A. Nawaz A. Naz S. Hussain S.. (2020). Role of osmolytes in the mechanisms of antioxidant defense of plants. Sustain. Agric. Rev. 39, 95–117. doi: 10.1007/978-3-030-38881-2_4

[ref29] FAOSTAT (2021). Available at: http://www.fao.org/faostat/en/#data/QC2017. Accessed March 21, 2021.

[ref30] Gantait S. Panigrahi J. Patel I. C. Labrooy C. Rathnakumar A. L. Yasin J. K. (2020). “Peanut (Arachis hypogaea l.) breeding,” in Advances in Plant Breeding Strategies: Nut and Beverage Crops. eds. Al-Khayri J. M. Jain S. M. Johnson D. V. (Cham: Springer), 253–299.

[ref31] Geng B. Wang Q. Huang R. Liu Y. Guo Z. Lu S. (2021). A novel LRR-RLK (CTLK) confers cold tolerance through regulation on the C-repeat-binding factor pathway, antioxidants, and proline accumulation. Plant J. 108, 1679–1689. doi: 10.1111/tpj.15535, PMID: 34626033

[ref32] Gong H. Rafi K. Starr T. Stucker B. (2013). Generation and detection of defects in metallic parts fabricated by selective laser melting and electron beam melting and their effects on mechanical properties. Solid Free. Fabr.

[ref33] Grimaud F. Renaut J. Dumont E. Sergeant K. Lucau-Danila A. Blervacq A. S.. (2013). Exploring chloroplastic changes related to chilling and freezing tolerance during cold acclimation of pea (Pisum sativum L.). J. Proteomics 80, 145–159. doi: 10.1016/j.jprot.2012.12.030, PMID: 23318888

[ref34] Guan S. Xu Q. Ma D. Zhang W. Xu Z. Zhao M.. (2019). Transcriptomics profiling in response to cold stress in cultivated rice and weedy rice. Gene 685, 96–105. doi: 10.1016/j.gene.2018.10.066, PMID: 30389557

[ref35] Hasanuzzaman M. Nahar K. Rahman A. Mahmud J. A. Hossain M. S. Fujita M. (2016). “4 - Soybean Production and Environmental Stresses,” Environmental Stresses in Soybean Production. ed. Miransari M. (Academic Press), 61–102.

[ref36] Hatfield J. L. Prueger J. H. (2015). Temperature extremes: effect on plant growth and development. Weather Clim. Extrem. 10, 4–10. doi: 10.1016/j.wace.2015.08.001

[ref37] He X. Z. Dixon R. A. (2000). Genetic manipulation of isoflavone 7-O-methyltransferase enhances biosynthesis of 4’-O-methylated isoflavonoid phytoalexins and disease resistance in alfalfa. Plant Cell 12, 1689–1702. doi: 10.1105/tpc.12.9.1689, PMID: 11006341 PMC149079

[ref38] Heidarvand L. Maali-Amiri R. (2013). Physio-biochemical and proteome analysis of chickpea in early phases of cold stress. J. Plant Physiol. 170, 459–469. doi: 10.1016/j.jplph.2012.11.021, PMID: 23395538

[ref39] Huang B. Chu C. H. Chen S. L. Juan H. F. Chen Y. M. (2006). A proteomics study of the mung bean epicotyl regulated by brassinosteroids under conditions of chilling stress. Cell. Mol. Biol. Lett. 11, 264–278. doi: 10.2478/s11658-006-0021-7, PMID: 16847571 PMC6275966

[ref40] Huang X. Chen M. H. Yang L. T. Li Y. R. Wu J. M. (2015). Effects of exogenous abscisic acid on cell membrane and endogenous hormone contents in leaves of sugarcane seedlings under cold stress. Sugar Tech 17, 59–64. doi: 10.1007/s12355-014-0343-0

[ref41] Inostroza L. Bhakta M. Acuña H. Vásquez C. Ibáñez J. Tapia G.. (2018). Understanding the complexity of cold tolerance in white clover using temperature gradient locations and a GWAS approach. Plant Genome 11:170096. doi: 10.3835/plantgenome2017.11.0096 PMC12810145 30512038

[ref42] Islam I. Adam Z. Islam S. (2019). Soybean (*Glycine Max*): alternative sources of human nutrition and bioenergy for the 21st century. Am. J. Food Sci. Technol. 7, 1–6. doi: 10.12691/ajfst-7-1-1

[ref43] Jaganathan D. Bohra A. Thudi M. Varshney R. K. (2020). Fine mapping and gene cloning in the post-NGS era: advances and prospects. Theor. Appl. Genet. 133, 1791–1810. doi: 10.1007/s00122-020-03560-w, PMID: 32040676 PMC7214393

[ref44] Jan N. Qazi H. A. Raja V. John R. (2019). Proteomics: a tool to decipher cold tolerance. Theor. Exp. Plant Physiol. 31, 183–213. doi: 10.1007/s40626-019-00140-2

[ref45] Jan N. Rather A. M.-U.-D. John R. Chaturvedi P. Ghatak A. Weckwerth W.. (2022). Proteomics for abiotic stresses in legumes: present status and future directions. Crit. Rev. Biotechnol., 1–20. doi: 10.1080/07388551.2021.2025033, PMID: 35109728

[ref134] Janas K. M. Cvikrová M. Pałagiewicz A. Szafranska K. Posmyk M. M. (2002). Constitutive elevated accumulation of phenylpropanoids in soybean roots at low temperature. Plant Sci., 163, 369–373. doi: 10.1016/S0168-9452(02)00136-X

[ref46] Jha U. C. Nayyar H. Jha R. Nath C. P. Datta D. (2020). Chickpea Breeding for Abiotic Stress: Breeding Tools and ‘Omics’ Approaches for Enhancing Genetic Gain. Accelerated Plant Breeding 3, 211–234. doi: 10.1007/978-3-030-47306-8_8

[ref47] Jiang C. Zhang H. Ren J. Dong J. Zhao X. Wang X.. (2020). Comparative transcriptome-based mining and expression profiling of transcription factors related to cold tolerance in peanut. Int. J. Mol. Sci. 21:1921. doi: 10.3390/ijms21061921, PMID: 32168930 PMC7139623

[ref48] Joshi R. Singh B. Chinnusamy V. (2018). “Genetically engineering cold stress-tolerant crops: approaches and challenges,” in Cold Tolerance in Plants. eds. Wani S. H. Herath V. (Cham: Springer).

[ref49] Kaplan F. Kopka J. Haskell D. W. Zhao W. Schiller K. C. Gatzke N.. (2004). Exploring the temperature-stress metabolome of Arabidopsis. Plant Physiol. 136, 4159–4168. doi: 10.1104/pp.104.052142, PMID: 15557093 PMC535846

[ref50] Karami-Moalem S. Maali-Amiri R. Kazemi-Shahandashti S. S. (2018). Effect of cold stress on oxidative damage and mitochondrial respiratory properties in chickpea. Plant Physiol. Biochem. 122, 31–39. doi: 10.1016/j.plaphy.2017.11.011, PMID: 29172103

[ref51] Kazemi-Shahandashti S. S. Maali-Amiri R. (2018). Global insights of protein responses to cold stress in plants: signaling, defence, and degradation. J. Plant Physiol. 226, 123–135. doi: 10.1016/j.jplph.2018.03.022, PMID: 29758377

[ref52] Kazemi-Shahandashti S. S. Maali-Amiri R. Zeinali H. Khazaei M. Talei A. Ramezanpour S. S. (2014). Effect of short-term cold stress on oxidative damage and transcript accumulation of defense-related genes in chickpea seedlings. J. Plant Physiol. 171, 1106–1116. doi: 10.1016/j.jplph.2014.03.020, PMID: 24972025

[ref53] Kazemi Shahandashti S. S. Maali Amiri R. Zeinali H. Ramezanpour S. S. (2013). Change in membrane fatty acid compositions and cold-induced responses in chickpea. Mol. Biol. Rep. 40, 893–903. doi: 10.1007/s11033-012-2130-x, PMID: 23065233

[ref54] Khan S. A. Li M. Z. Wang S. M. Yin H. J. (2018). Revisiting the role of plant transcription factors in the battle against abiotic stress. Int. J. Mol. Sci. 19. doi: 10.3390/ijms19061634 PMC6032162 29857524

[ref55] Kidokoro S. Watanabe K. Ohori T. Moriwaki T. Maruyama K. Mizoi J.. (2015). Soybean DREB1/CBF-type transcription factors function in heat and drought as well as cold stress-responsive gene expression. Plant J. 81, 505–518. doi: 10.1111/tpj.12746, PMID: 25495120

[ref56] Kim Y. S. Lee M. Lee J. H. Lee H. J. Park C. M. (2015). The unified ICE–CBF pathway provides a transcriptional feedback control of freezing tolerance during cold acclimation in Arabidopsis. Plant Mol. Biol. 89, 187–201. doi: 10.1007/s11103-015-0365-3, PMID: 26311645

[ref57] Klein A. Houtin H. Rond C. Marget P. Jacquin F. Boucherot K.. (2014). QTL analysis of frost damage in pea suggests different mechanisms involved in frost tolerance. Theor. Appl. Genet. 127, 1319–1330. doi: 10.1007/s00122-014-2299-6, PMID: 24695842

[ref003] Knoll J. (2008). QTL analysis of early-season cold tolerance in sorghum. TAG. Theoretical and applied genetics. Theor. Appl. Genet. 116, 577–587. 18097644 10.1007/s00122-007-0692-0

[ref129] Kosová K. Vítámvás P. Prášil I. T. Klíma M. Renaut J. (2021). Plant Proteoforms Under Environmental Stress: Functional Proteins Arising From a Single Gene. Front Plant Sci. 12:793113. doi: 10.3389/fpls.2021.793113 34970290 PMC8712444

[ref58] Kumar A. Dixit S. Ram T. Yadaw R. B. Mishra K. K. Mandal N. P. (2014). Breeding high-yielding drought-tolerant rice: genetic variations and conventional and molecular approaches. J. Exp. Bot. 65, 6265–6278. doi: 10.1093/jxb/eru363, PMID: 25205576 PMC4223988

[ref59] Kumar S. Nayyar H. Bhanwara R. Upadhyaya H. (2010). Chilling stress effects on reproductive biology of chickpea. J. SAT Agric. Res. 8, 1–14.

[ref60] Larance M. Lamond A. I. (2015). Multidimensional proteomics for cell biology. Nat. Rev. Mol. Cell Biol. 16, 269–280. doi: 10.1038/nrm3970 25857810

[ref61] Levitt J. (1980). Responses of Plants to Environmental Stress, Volume 1: *Chilling, Freezing, and High Temperature Stresses*. Cambridge: Academic Press.

[ref62] Li C. Sun B. Li Y. Liu C. Wu X. Zhang D.. (2016). Numerous genetic loci identified for drought tolerance in the maize nested association mapping populations. BMC Genomics 17:894. doi: 10.1186/s12864-016-3170-8, PMID: 27825295 PMC5101730

[ref63] Lyu J. I. Ramekar R. Kim J. M. Hung N. N. Seo J. S. Kim J. B.. (2021). Unraveling the complexity of faba bean (Vicia faba L.) transcriptome to reveal cold-stress-responsive genes using long-read isoform sequencing technology. Sci. Rep. 11:21094. doi: 10.1038/s41598-021-00506-0, PMID: 34702863 PMC8548339

[ref006] Macas J. Novak P. Pellicer J. Čížková J. Koblížková A. Neumann P.. (2015). In depth characterization of repetitive DNA in 23 plant genomes reveals sources of genome size variation in the legume tribe Fabeae. PLoS One 10:e0143424. doi: 10.1371/journal.pone.0143424, PMID: 26606051 PMC4659654

[ref64] Maqbool A. Shafiq S. Lake L. (2010). Radiant frost tolerance in pulse crops-a review. Euphytica 172, 1–12. doi: 10.1007/s10681-009-0031-4

[ref132] Mecha E. Erny G. L. Guerreiro A. C. L. Feliciano R. P. Barbosa I. da Silva A. B.. (2022). Metabolomics profile responses to changing environments in a common bean (Phaseolus vulgaris L.) germplasm collection. Food Chem., 370:131003. doi: 10.1016/j.foodchem.2021.131003 34543920

[ref65] Meena R. S. Lal R. (2018). “Legumes and sustainable use of soils,” in Legumes for Soil Health and Sustainable Management. eds. Meena R. S. Das A. Yadav G. S. Lal R. (Singapore: Springer)

[ref66] Miao Z. Xu W. Li D. Hu X. Liu J. Zhang R.. (2015). De novo transcriptome analysis of Medicago falcata reveals novel insights about the mechanisms underlying abiotic stress-responsive pathway. BMC Genomics 16:818. doi: 10.1186/s12864-015-2019-x, PMID: 26481731 PMC4615886

[ref67] Minton K. (2016). Gene expression: reading protein acetylation. Nat. Rev. Mol. Cell Biol. 17, 676–677. doi: 10.1038/nrm.2016.137 27703241

[ref68] Mortazavi A. Williams B. A. McCue K. Schaeffer L. Wold B. (2008). Mapping and quantifying mammalian transcriptomes by RNA-Seq. Nat. Methods 5, 621–628. doi: 10.1038/nmeth.1226, PMID: 18516045 PMC13303166

[ref69] Mugabe D. Coyne C. J. Piaskowski J. Zheng P. Ma Y. Landry E.. (2019). Quantitative trait loci for cold tolerance in Chickpea. Crop Sci. 59, 573–582. doi: 10.2135/cropsci2018.08.0504

[ref70] Murata N. Sato N. Takahashi N. Hamazaki Y. (1982). Compositions and positional distributions of fatty acids in phospholipids from leaves of chilling-sensitive and chilling-resistant plants. Plant Cell Physiol. 23, 1071–1079. doi: 10.1093/oxfordjournals.pcp.a076437

[ref71] Nagalakshmi U. Wang Z. Waern K. Shou C. Raha D. Gerstein M.. (2008). The transcriptional landscape of the yeast genome defined by RNA sequencing. Science 320, 1344–1349. doi: 10.1126/science.1158441, PMID: 18451266 PMC2951732

[ref72] Nayyar H. Bains T. S. Kumar S. Kaur G. (2005). Chilling effects during seed filling on accumulation of seed reserves and yield of chickpea. J. Sci. Food Agric. 85, 1925–1930. doi: 10.1002/jsfa.2198

[ref73] Ng D. W. K. Abeysinghe J. K. Kamali M. (2018). Regulating the regulators: the control of transcription factors in plant defense signaling. Int. J. Mol. Sci. 19:3737. doi: 10.3390/ijms19123737, PMID: 30477211 PMC6321093

[ref74] Nie J. Wen C. Xi L. Lv S. Zhao Q. Kou Y.. (2018). The AP2/ERF transcription factor CmERF053 of chrysanthemum positively regulates shoot branching, lateral root, and drought tolerance. Plant Cell Rep. 37, 1049–1060. doi: 10.1007/s00299-018-2290-9, PMID: 29687169

[ref75] Novák A. Boldizsár Á. Ádám É. Kozma-Bognár L. Majláth I. Båga M.. (2016). Light-quality and temperature-dependent CBF14 gene expression modulates freezing tolerance in cereals. J. Exp. Bot. 67, 1285–1295. doi: 10.1093/jxb/erv526, PMID: 26712822

[ref76] Öktem H. A. Eyidodan F. Demirba D. Bayraç A. T. Öz M. T. Özgür E.. (2008). Antioxidant responses of lentil to cold and drought stress. J. Plant Biochem. Biotechnol. 17, 15–21. doi: 10.1007/BF03263254

[ref77] Oliver S. N. Van Dongen J. T. Alfred S. C. Mamun E. A. Zhao X. Saini H. S.. (2005). Cold-induced repression of the rice anther-specific cell wall invertase gene OSINV4 is correlated with sucrose accumulation and pollen sterility. Plant, Cell Environ. 28, 1534–1551. doi: 10.1111/j.1365-3040.2005.01390.x

[ref78] Pandey H. Singh D. Pandey A. K. Suthar K. P. Mehta R. Pandey D. (2021). “Current approaches in horticultural crops to mitigate the effect of cold stress,” in Stress Tolerance in Horticultural Crops. eds. Kumar A. Rai A. C. Rai A. Rai K. K. Rai V. P. (Sawston: Woodhead Publishing).

[ref79] Pandey P. Ramegowda V. Senthil-Kumar M. (2015). Shared and unique responses of plants to multiple individual stresses and stress combinations: physiological and molecular mechanisms. Front. Plant Sci. 6:723. doi: 10.3389/fpls.2015.00723, PMID: 26442037 PMC4584981

[ref80] Pang T. Ye C. Y. Xia X. Yin W. (2013). De novo sequencing and transcriptome analysis of the desert shrub, Ammopiptanthus mongolicus, during cold acclimation using Illumina/Solexa. BMC Genomics 14, 1–5. doi: 10.1186/1471-2164-14-488, PMID: 23865740 PMC3728141

[ref81] Pareddy D. Chennareddy S. Anthony G. Sardesai N. Mall T. Minnicks T.. (2020). Improved soybean transformation for efficient and high throughput transgenic production. Transgenic Res. 29, 267–281. doi: 10.1007/s11248-020-00198-8, PMID: 32303980

[ref136] Patel J. Khandwal D. Choudhary B. Ardeshana D. Jha R. K. Tanna B.. (2022). Differential physio-biochemical and metabolic responses of peanut (Arachishypogaea L.) under multiple abiotic stress conditions. Int. J. Mol. Sci. 23:660. doi: 10.3390/ijms23020660 35054846 PMC8776106

[ref82] Puli C. O. R. Akila C. S. Pandit V. Konduru S. Kandi S. R. Chinta S. (2021). Peanut (*Arachis hypogaea* L.) Transgenic Plants for Abiotic Stress Tolerance. in Genetically Modified Crops.

[ref83] Rakei A. Maali-Amiri R. Zeinali H. Ranjbar M. (2016). DNA methylation and physio-biochemical analysis of chickpea in response to cold stress. Protoplasma 253, 61–76. doi: 10.1007/s00709-015-0788-3, PMID: 25820678

[ref84] Ramalingam A. Kudapa H. Pazhamala L. T. Weckwerth W. Varshney R. K. (2015). Proteomics and metabolomics: two emerging areas for legume improvement. Front. Plant Sci. 6:1116. doi: 10.3389/fpls.2015.01116, PMID: 26734026 PMC4689856

[ref85] Repo T. Mononen K. Alvila L. Pakkanen T. T. Hänninen H. (2008). Cold acclimation of pedunculate oak (*Quercus robur* L.) at its northernmost distribution range. Environ. Exp. Bot. 63, 59–70. doi: 10.1016/j.envexpbot.2007.10.023

[ref86] Rodziewicz P. Swarcewicz B. Chmielewska K. Wojakowska A. Stobiecki M. (2014). Influence of abiotic stresses on plant proteome and metabolome changes. Acta Physiol. Plant. 36, 1–19. doi: 10.1007/s11738-013-1402-y

[ref004] Sallam A. Arbaoui M. El-Esawi M. Abshire N. Martsch R. (2016). Identification and verification of QTL associated with frost tolerance using linkage mapping and GWAS in winter faba bean. Front. Plant Sci. 7:1098. doi: 10.3389/fpls.2016.01098 27540381 PMC4972839

[ref87] Sanghera G. S. Wani S. H. Hussain W. Singh N. B. (2011). Engineering cold stress tolerance in crop plants. Curr. Genomics 12, 30–43. doi: 10.2174/138920211794520178, PMID: 21886453 PMC3129041

[ref88] Sansone S. A. Fan T. Goodacre R. Griffin J. L. Hardy N. W. Kaddurah-Daouk R.. (2007). The metabolomics standards initiative [3]. Nat. Biotechnol. 25, 846–848. doi: 10.1038/nbt0807-846b 17687353

[ref008] Sato S. Nakamura Y. Kaneko T. Asamizu E. Kato T. Nakao M.. (2008). Genome structure of the legume, Lotus japonicus. DNA research: an international journal for rapid publication of reports on genes and genomes 15, 227–239. doi: 10.1093/dnares/dsn008 18511435 PMC2575887

[ref007] Schmutz J. Cannon S. B. Schlueter J. Ma J. Mitros T. Nelson W.. (2010). Genome sequence of the paleopolyploid soybean. Nature 463, 178–183. 20075913 10.1038/nature08670

[ref89] Shafiq M. Z. Ji L. Liu A. X. Pang J. Wang J. (2012). A first look at cellular machine-to-machine traffic - large scale measurement and characterization. Perform. Eval. Rev. 40, 65–76.

[ref90] Sharma K. D. Nayyar H. (2014). Cold stress alters transcription in meiotic anthers of cold tolerant chickpea (*Cicer arietinum* L.). BMC Res. Notes 7:7. doi: 10.1186/1756-0500-7-717, PMID: 25306382 PMC4201710

[ref91] Sharma K. K. Ortiz R. (2000). Program for the application of genetic transformation for crop improvement in the semi-arid tropics. Vitr. Cell. Dev. Biol. - Plant 36, 83–92. doi: 10.1007/s11627-000-0019-1

[ref92] Sharma K. Lavanya M. (2002). Recent developments in transgenics for abiotic stress in legumes of the semi-arid tropics. JIRCAS Work. Rep. No. 23:23

[ref93] Sharma L. Priya M. Bindumadhava H. Nair R. M. Nayyar H. (2016). Influence of high temperature stress on growth, phenology and yield performance of mungbean [*Vigna radiata* (L.) Wilczek] under managed growth conditions. Sci. Hortic. 213, 379–391. doi: 10.1016/j.scienta.2016.10.033

[ref94] Sharma P. Sharma N. Deswal R. (2005). The molecular biology of the low-temperature response in plants. BioEssays 27, 1048–1059. doi: 10.1002/bies.20307 16163711

[ref95] Sharmila P. Phanindra M. L. V. Anwar F. Singh K. Gupta S. Pardha Saradhi P. (2009). Targeting prokaryotic choline oxidase into chloroplasts enhance the potential of photosynthetic machinery of plants to withstand oxidative damage. Plant Physiol. Biochem. 47, 391–396. doi: 10.1016/j.plaphy.2009.01.001, PMID: 19186067

[ref96] Shi H. He S. He X. Lu S. Guo Z. (2019). An eukaryotic elongation factor 2 from Medicago falcata (MfEF2) confers cold tolerance. BMC Plant Biol. 19:218. doi: 10.1186/s12870-019-1826-7, PMID: 31133003 PMC6537394

[ref97] Singh N. K. Gupta D. K. Jayaswal P. K. Mahato A. K. Dutta S. Singh S.. (2012). The first draft of the pigeonpea genome sequence. J. Plant Biochem. Biotechnol. 21, 98–112. doi: 10.1007/s13562-011-0088-8, PMID: 24431589 PMC3886394

[ref98] Singhal R. Mitta S. R. Das N. K. Kerk S. A. Sajjakulnukit P. Solanki S.. (2021). HIF-2α activation potentiates oxidative cell death in colorectal cancers by increasing cellular iron. J. Clin. Invest. 131. doi: 10.1172/JCI143691, PMID: 33914705 PMC8203462

[ref99] Sinha S. Kukreja B. Arora P. Sharma M. Pandey G. K. Agarwal M.. (2015). The omics of cold stress responses in plants. in Elucidation of Abiotic Stress Signaling in Plants: Functional Genomics Perspectives, Volume 2, The Omics of Cold Stress Responses in Plants.

[ref100] Song L. Jiang L. Chen Y. Shu Y. Bai Y. Guo C. (2016). Deep-sequencing transcriptome analysis of field-grown Medicago sativa L. crown buds acclimated to freezing stress. Funct. Integr. Genomics 16, 495–511. doi: 10.1007/s10142-016-0500-5, PMID: 27272950

[ref101] Srivastava R. Sahoo L. (2021). Balancing yield trade-off in legumes during multiple stress tolerance via strategic crosstalk by native NAC transcription factors. J. Plant Biochem. Biotechnol. 30, 708–729. doi: 10.1007/s13562-021-00749-y

[ref102] Sun X. Wang Y. Sui N. (2018). Transcriptional regulation of bHLH during plant response to stress. Biochem. Biophys. Res. Commun. 503, 397–401. doi: 10.1016/j.bbrc.2018.07.123 30057319

[ref103] Suzuki K. Miki S. Shiki S. Wang Z. Ohkubo M. (2008). Time resolution improvement of superconducting NbN stripline detectors for time-of-flight mass spectrometry. Appl. Phys. Express 1:031702. doi: 10.1143/APEX.1.031702

[ref104] Swigonska S. Weidner S. (2013). Proteomic analysis of response to long-term continuous stress in roots of germinating soybean seeds. J. Plant Physiol. 170, 470–479. doi: 10.1016/j.jplph.2012.11.020, PMID: 23394790

[ref105] Tan H. Huang H. Tie M. Tang Y. Lai Y. Li H. (2016). Transcriptome profiling of two asparagus bean (Vigna unguiculata subsp. sesquipedalis) cultivars differing in chilling tolerance under cold stress. PLoS One 11:11. doi: 10.1371/journal.pone.0151105, PMID: 26954786 PMC4783050

[ref106] Taylor N. L. Heazlewood J. L. Day D. A. Millar A. H. (2005). Differential impact of environmental stresses of the pea mitochondrial proteome. Mol. Cell. Proteomics 4, 1122–1133. doi: 10.1074/mcp.M400210-MCP200, PMID: 15914488

[ref107] Tesfaye M. Temple S. J. Allan D. L. Vance C. P. Samac D. A. (2001). Overexpression of malate dehydrogenase in transgenic alfalfa enhances organic acid synthesis and confers tolerance to aluminum. Plant Physiol. 127, 1836–1844. doi: 10.1104/pp.010376, PMID: 11743127 PMC133587

[ref108] Tian X. Liu Y. Huang Z. Duan H. Tong J. He X.. (2015). Comparative proteomic analysis of seedling leaves of cold-tolerant and -sensitive spring soybean cultivars. Mol. Biol. Rep. 42, 581–601. doi: 10.1007/s11033-014-3803-4, PMID: 25359310

[ref005] Upadhyaya H. D. Wang Y. H. Sastry D. V. Dwivedi S. L. Prasad P. V. Burrell A. M.. (2016). Association mapping of germinability and seedling vigor in sorghum under controlled low-temperature conditions. Genome 59, 137–145. doi: 10.1139/gen-2015-0122, PMID: 26758024

[ref109] Vara Prasad P. V. Craufurd P. Q. Summerfield R. J. Wheeler T. R. (2000). Effects of short episodes of heat stress on flower production and fruit-set of groundnut (Arachis hypogaea L.). J. Exp. Bot. 51, 777–784. doi: 10.1093/jexbot/51.345.777, PMID: 10938870

[ref110] Varshney R. K. Pandey M. K. Bohra A. Singh V. K. Thudi M. Saxena R. K. (2019). Toward the sequence-based breeding in legumes in the post-genome sequencing era. Theor. Appl. Genet. 132, 797–816. doi: 10.1007/s00122-018-3252-x, PMID: 30560464 PMC6439141

[ref111] Varshney R. K. Thudi M. Nayak S. N. Gaur P. M. Kashiwagi J. Krishnamurthy L.. (2014). Genetic dissection of drought tolerance in chickpea (Cicer arietinum L.). Theor. Appl. Genet. 127, 445–462. doi: 10.1007/s00122-013-2230-6, PMID: 24326458 PMC3910274

[ref112] Wang T. L. Domoney C. Hedley C. L. Casey R. Grusak M. A. (2003). Can we improve the nutritional quality of legume seeds? Plant Physiol. 131, 886–891. doi: 10.1104/pp.102.017665, PMID: 12644641 PMC1540288

[ref113] Wang W. Gao T. Chen J. Yang J. Huang H. Yu Y. (2019). The late embryogenesis abundant gene family in tea plant (Camellia sinensis): genome-wide characterization and expression analysis in response to cold and dehydration stress. Plant Physiol. Biochem. 135, 277–286. doi: 10.1016/j.plaphy.2018.12.009, PMID: 30593000

[ref114] Wang X. Liu Y. Han Z. Chen Y. Huai D. Kang Y.. (2021). Integrated transcriptomics and metabolomics analysis reveal key metabolism pathways contributing to cold tolerance in peanut. Front. Plant Sci. 12:752474. doi: 10.3389/fpls.2021.752474, PMID: 34899780 PMC8652294

[ref115] Wani S. H. Sah S. K. Sanghera G. Hussain W. Singh N. B. (2016). Genetic engineering for cold stress tolerance in crop plants. Adv. Genome Sci. 4, 173–201. doi: 10.2174/9781681081731116040010

[ref116] Weckwerth P. Ehlert B. Romeis T. (2015). ZmCPK1, a calcium-independent kinase member of the Zea maysCDPK gene family, functions as a negative regulator in cold stress signalling. Plant Cell Environ. 38, 544–558. doi: 10.1111/pce.12414 25052912

[ref117] Weckwerth W. (2011). Green systems biology - from single genomes, proteomes and metabolomes to ecosystems research and biotechnology. J. Proteomics 75, 284–305. doi: 10.1016/j.jprot.2011.07.010, PMID: 21802534

[ref118] Wilhelm B. T. Marguerat S. Watt S. Schubert F. Wood V. Goodhead I.. (2008). Dynamic repertoire of a eukaryotic transcriptome surveyed at single-nucleotide resolution. Nature 453, 1239–1243. doi: 10.1038/nature07002, PMID: 18488015

[ref119] Wu Q. VanEtten H. D. (2004). Introduction of plant and fungal genes into pea (Pisum sativum L.) hairy roots reduces their ability to produce pisatin and affects their response to a fungal pathogen. Mol. Plant-Microbe Interact. 17, 798–804. doi: 10.1094/MPMI.2004.17.7.798, PMID: 15242174

[ref120] Xiaoqin W. Pingfang Y. Xiaofeng Z. Yinong X. Tingyun K. Shihua S.. (2009). Proteomic analysis of the cold stress response in the moss, Physcomitrella patens. Proteomics 9, 4529–4538. doi: 10.1002/pmic.200900062, PMID: 19670371

[ref121] Xu G. Singh S. K. Reddy V. R. Barnaby J. Y. Sicher R. C. Li T. (2016). Soybean grown under elevated CO2 benefits more under low temperature than high temperature stress: varying response of photosynthetic limitations, leaf metabolites, growth, and seed yield. J. Plant Physiol. 205, 20–32. doi: 10.1016/j.jplph.2016.08.003, PMID: 27589223

[ref133] Yeshi K. Crayn D. Ritmejerytė E. Wangchuk P. (2021). Plant Secondary Metabolites Produced in Response to Abiotic Stresses Has Potential Application in Pharmaceutical Product Development. Molecules (Switzerland: Basel), 27:313. 10.3390/molecules27010313 PMC8746929 35011546

[ref122] Zargar S. M. Mahajan R. Nazir M. Nagar P. Kim S. T. Rai V.. (2017). Common bean proteomics: Present status and future strategies. J. Proteomics 169, 239–248. doi: 10.1016/j.jprot.2017.03.019, PMID: 28347863

[ref123] Zhang H. Dong J. Zhao X. Zhang Y. Ren J. Xing L.. (2019). Research progress in membrane lipid metabolism and molecular mechanism in peanut cold tolerance. Front. Plant Sci. 10:838. doi: 10.3389/fpls.2019.00838, PMID: 31316538 PMC6610330

[ref124] Zhang T. Mo J. Zhou K. Chang Y. Liu Z. (2018). Overexpression of Brassica campestris BcICE1 gene increases abiotic stress tolerance in tobacco. Plant Physiol. Biochem. 132, 515–523. doi: 10.1016/j.plaphy.2018.09.039, PMID: 30312954

[ref125] Zhang X. H. Wei Q. Z. Widholm J. M. (2005). Expression of a fungal cyanamide hydratase in transgenic soybean detoxifies cyanamide in tissue culture and in planta to provide cyanamide resistance. J. Plant Physiol. 162, 1064–1073. doi: 10.1016/j.jplph.2004.11.013, PMID: 16173468

[ref126] Zhou P. Li Q. Liu G. Xu N. Yang Y. Zeng W.. (2019). Integrated analysis of transcriptomic and metabolomic data reveals critical metabolic pathways involved in polyphenol biosynthesis in Nicotiana tabacum under chilling stress. Funct. Plant Biol. 46, 30–43. doi: 10.1071/FP18099, PMID: 30939256

[ref127] Zhuo C. Liang L. Zhao Y. Guo Z. Lu S. (2018). A cold responsive ethylene responsive factor from Medicago falcata confers cold tolerance by up-regulation of polyamine turnover, antioxidant protection, and proline accumulation. Plant Cell Environ. 41, 2021–2032. doi: 10.1111/pce.13114, PMID: 29216408

[ref128] Zhuo C. Wang T. Guo Z. Lu S. (2016). Overexpression of MfPIP2-7 from Medicago falcata promotes cold tolerance and growth under NO3- deficiency in transgenic tobacco plants. BMC Plant Biol. 16:138. doi: 10.1186/s12870-016-0814-4, PMID: 27301445 PMC4907284

